# Ribosome Inactivating Proteins from Rosaceae

**DOI:** 10.3390/molecules21081105

**Published:** 2016-08-22

**Authors:** Chenjing Shang, Pierre Rougé, Els J. M. Van Damme

**Affiliations:** 1 Department of Molecular Biotechnology, Faculty of Bioscience Engineering, Ghent University, 9000 Ghent, Belgium; shangchenjing@scsio.ac.cn; 2 Unité Mixte de Recherche 152 Pharma Développement, Institut de Recherche pour le Développement, Université Paul Sabatier, 31062 Toulouse, France; pierre.rouge@free.fr

**Keywords:** carbohydrate binding activity, molecular modeling, protein synthesis inhibition, ribosome-inactivating proteins

## Abstract

Ribosome-inactivating proteins (RIPs) are widespread among higher plants of different taxonomic orders. In this study, we report on the RIP sequences found in the genome/transcriptome of several important Rosaceae species, including many economically important edible fruits such as apple, pear, peach, apricot, and strawberry. All RIP domains from Rosaceae share high sequence similarity with conserved residues in the catalytic site and the carbohydrate binding sites. The genomes of *Malus domestica* and *Pyrus communis* contain both type 1 and type 2 RIP sequences, whereas for *Prunus mume*, *Prunus persica*, *Pyrus bretschneideri*, and *Pyrus communis* a complex set of type 1 RIP sequences was retrieved. Heterologous expression and purification of the type 1 as well as the type 2 RIP from apple allowed to characterize the biological activity of the proteins. Both RIPs from *Malus domestica* can inhibit protein synthesis. Furthermore, molecular modelling suggests that RIPs from Rosaceae possess three-dimensional structures that are highly similar to the model proteins and can bind to RIP substrates. Screening of the recombinant type 2 RIP from apple on a glycan array revealed that this type 2 RIP interacts with terminal sialic acid residues. Our data suggest that the RIPs from Rosaceae are biologically active proteins.

## 1. Introduction

Ribosome-inactivating proteins (RIPs) are a large family of enzymes (EC.3.2.2.22) comprising an rRNA *N*-glycosylase domain that is capable of catalytically inactivating ribosomes through the removal of a specific adenine residue from a highly conserved α-sarcin/ricin loop within the large rRNA [1]. Though RIPs have first been detected and characterized from plants, RIPs have also been isolated and characterized from bacteria and fungi, and more recently RIP sequences have also been reported in insects [2]. However, apart from the shiga and shiga-like toxins from bacteria [3] and a few fungal RIPs from mushrooms [4], virtually all research concentrated on RIPs are from flowering plants. Plant RIPs are classically subdivided in two major groups: Type 1 RIPs consist of a single protein domain with rRNA *N*-glycosylase activity (RIP domain), whereas type 2 RIPs are chimeric proteins built up of an *N*-terminal rRNA *N*-glycosylase domain (RIP domain) fused to a C-terminal carbohydrate binding domain (lectin domain).

RIPs are widely distributed in the plant kingdom and RIP sequences have been reported for at least 71 monocotyledonous and dicotyledonous species within the Angiospermae [5]. However, RIP sequences are not ubiquitous in plants. For example, no RIP sequence could be retrieved from the complete genome of *Arabidopsis thaliana* [6]. At present, RIPs have been reported frequently in the plant families Cucurbitaceae, Euphorbiaceae, Sambucaceae, Phytolaccaceae, Poaceae, and Caryophyllaceae [7]. RIPs are not associated with (a) particular tissue(s) but are found in virtually all plant parts (e.g., seeds, roots, leaves, bulbs, fruits, and bark) [8]. Both the distribution over different tissues and the abundance are highly variable depending on the species. Though multiple RIP sequences have been reported within one species, usually most of these sequences belong to the same class of RIP proteins. Several type 1 RIP isoforms have been reported in *Phytolacca americana*, referred to as pokeweed antiviral protein or PAP [9]. Different isoforms can occur within the same tissue and with differential expression during development. For instance, PAP-I, PAP-II, and PAP-III are isolated from spring, early summer, and late summer leaves of *Phytolacca*. At least 31 members of the RIP family have been identified in the rice genome, all of them can be grouped as type 1 RIPs [10]. At present, only a few examples are known of plant species containing both type 1 and type 2 RIP sequences, such as e.g., *Iris x hollandica* [11], *Sambucus* sp. [12], *Momordica charantia* [13,14], and *Trichosanthes kirilowii* [15,16].

Most RIPs identified and studied today are expressed at high level which enabled the purification of the protein and characterization of their activities. Since more genome/transcriptome sequences have become available for plants in the last decades, these data also yielded more information with respect to the distribution and evolution of RIP sequences [5,6,17,18]. At present, it is clear that the distribution of RIPs is underestimated, since many RIPs are expressed at levels that are too low to allow purification of the protein.

Recently, the genome sequences of four Rosaceae species (*Malus domestica*, *Pyrus communis*, *Prunus persica*, and *Fragaria vesca*) have become public through the Genome Rosaceae Database. In silico analyses of these plant genomes/transcriptomes not only allowed identifying novel types of RIPs but yielded also a fairly detailed overview of the occurrence of the type 1 and type 2 RIPs in Rosaceae and generated new insights in the molecular evolution of this protein family. In this study, a detailed phylogenetic analysis of RIP sequences within Rosaceae has been performed. *Malus domestica* was selected as a model since it contains both type 1 and type 2 RIP sequences, further referred to as Md1RIP and Md2RIP, respectively. Detailed analyses of the RIP sequences combined with molecular modeling of the active site pocket of the RIP domain and the carbohydrate binding activity of the lectin domain provide the first structural information for Rosaceae RIPs.

## 2. Results

### 2.1. RIP Genes Are Present in Most Rosaceae Genomes

The presence and distribution of RIP sequences within the family Rosaceae was analyzed using BLAST searches against the genomes of all Rosaceae species for which a complete genome sequence is available. A total of 16 genes encoding putative type 1 RIP genes and two genes encoding putative type 2 RIP genes were identified in Rosaceae species including *Malus domestica* (three type 1 RIPs and one type 2 RIP), *Prunus mume* (four type 1 RIPs), *Prunus persica* (one type 1 RIP), *Pyrus bretschneideri* (four type 1 RIPs) and *Pyrus communis* (three type 1 RIPs and one type 2 RIP) (Figure S1 and Table S1 in the Supplementary Materials). Overall, the RIP sequences from Rosaceae show 55%–70% sequence similarity and 42%–60% sequence identity at amino acid level. Furthermore BLAST searches in the NCBI sequence database revealed that RIP genes from Rosaceae species share 37%–50% sequence identity with some well-characterized RIP domains, such as trichosanthin (type 1 RIP from *Trichosanthes kirilowii*) and SNA-I, ebulin, and cinnamomin (type 2 RIPs from *Sambucus nigra*, *Sambucus ebulus*, and *Cinnamonum camphora*, respectively).

**Figure 1:**
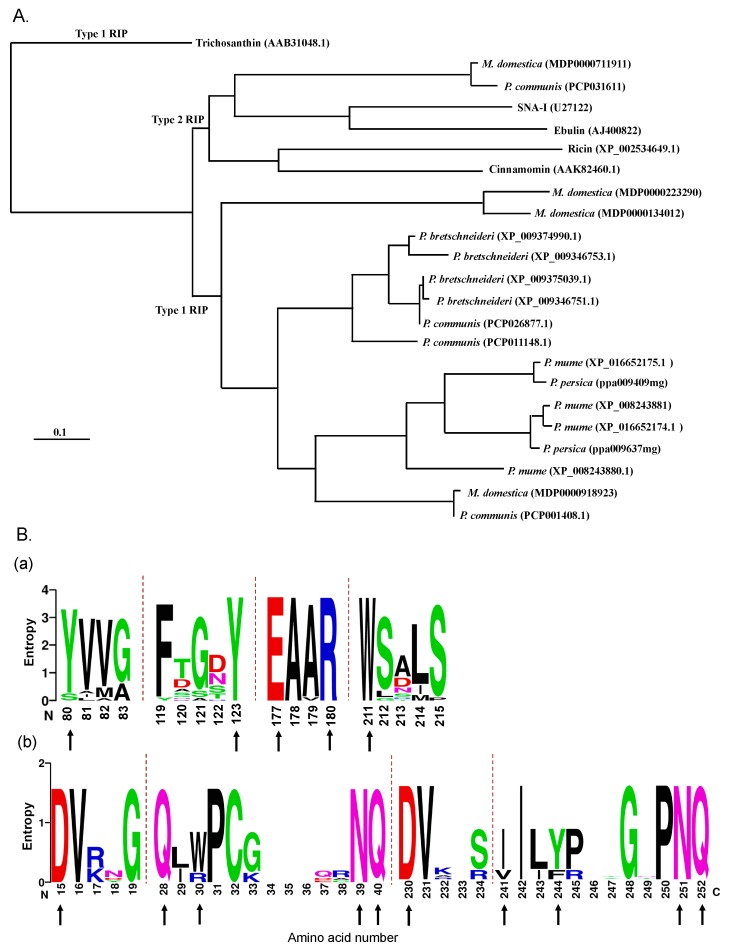
Sequence analyses of RIPs. (**A**) Phylogenetic tree of sequences encoding RIP domains from Rosaceae, *Ricinus communis*, *Sambucus nigra*, *Sambucus ebulus*, *Cinnamomum camphora*, and *Trichosanthes kirilowii*. The phylogenetic tree was made by using constraint-based alignment tool (COBALT). The name and species of Rosaceae RIPs and accession numbers are indicated; (**B**) (a) Logo representation of the amino acid sequence alignment of RIP domains presented in the phylogenetic tree. The size of the letters is proportional to the frequency of the amino acid at that position of the sequence. Residues reported to be important for the formation of active sites are indicated by black arrows [19]. Amino acid numbers refer to the sequence of ricin; (b) Amino acid conservation in the lectin domains of type 2 RIPs from *Malus domestica*, *Pyrus communis*, *Sambucus nigra*, *Sambucus ebulus*, and *Ricinus communis*. The conserved amino acids of the carbohydrate binding site are indicated by black arrows [20].

All Rosaceae genomes studied contained one or more sequences with a RIP domain, except for the genome of *Fragaria vesca* and *Fragaria ananassa*. Interestingly the genomes from *Malus domestica* (apple) and *Pyrus communis* (pear) contain both type 1 RIP and type 2 RIP sequences. To investigate the evolutionary relationships between the Rosaceae RIPs a phylogenetic analysis was performed for all RIP domains identified within Rosaceae (Figure 1A, Figure S1 in the Supplementary Materials). The dendrogram revealed two major clades (Figure 1A). The largest clade contains the RIP domain sequences for all type 1 RIPs from Rosaceae and falls apart in three subgroups with apple sequences, pear sequences, and a group containing sequences from *Malus*, *Pyrus*, and *Prunus*. The smaller clade clusters the RIP domains belonging to the type 2 RIPs from apple and pear. The RIP domain sequences of some well-characterized type 2 RIPs (ricin, SNA-I, ebulin and cinnamomin) with high sequence similarity to Rosaceae RIPs cluster in the same clade as the type 2 RIP domains from *Malus* and *Pyrus*. The sequence for the type 1 RIP trichosanthin forms a separate branch of the dendrogram (Figure 1A).

Sequence alignments for all RIP domains used in the phylogenetic tree were performed using ClustalW and analyzed for conserved positions in the amino acid sequences. The sequence logo representation is shown in Figure 1B (panel a) and highlights those amino acid residues that are highly conserved at several positions within the RIP domain sequence. 53 residues out of 277 (=average size of the RIP domain sequence) are conserved in at least 80% of the sequences analyzed. Five amino acids: Y80 (position numbering according to ricin sequence), Y123, E177, R180, and W211 are known to be important for the catalytic activity of the model RIP ricin [19] and are highly conserved for all the Rosaceae RIP domain sequences. Amino acid residues E177 and R180 are directly involved in the activity of catalytic sites, whereas Y80, Y123, and W211 play an important role in binding of the target adenine [5]. In type 2 RIP sequences from apple and pear residue Y80 is replaced by S71 (Figure 1B and Figure S2 in the Supplementary Materials).

A sequence logo representation for the lectin domain sequences of ricin, ebulin, SNA-I, and the type 2 RIPs from *M. domestica* and *P. communis* (Figure 1B panel b) showed that 149 residues are highly conserved in the sequences analyzed. Among them, 10 amino acid residues (D15, Q28, W30, N39, Q40, D230, I241, Y244, N251, Q252, position numbering according to ricin sequence) from Rosaceae type 2 RIPs are identical with the residues forming the carbohydrate binding sites of ricin.

### 2.2. Identification and Sequence Analysis of RIP Genes in the Malus Domestica Genome

The in silico analysis allowed identifying the RIP sequences in most Rosaceae genomes. However, these sequence data do not allow the prediction of the biochemical activities of the proteins. Since *Malus domestica* (apple) is one of the most important fruits, it was selected as a model to study the recombinant RIPs and characterize their biological activities.

The apple genome contains three type 1 RIP sequences and one type 2 RIP sequence. In addition, the screening of the apple genome also revealed the occurrence of several pseudogenes. Analysis of the apple type 1 RIP (Md1RIP) genes indicated that they encode three closely related proteins (Genome database for Rosaceae (GRD) accession no. MDP0000918923, MDP0000223290, MDP0000134012 further referred to as Md1RIP genes A, B and C, respectively) (Figure 1A). All Md1RIP sequences are highly similar with 48% sequence identity and 63%–64% sequence similarity. The Md1RIP sequences share the highest sequence identity with the RIP domain from cinnamomin (isoform iii) (43% sequence identity and 58% sequence similarity). None of these Md1RIP sequences are synthesized with a signal peptide (Figure S2 in the Supplementary Materials). Residues that build up the core catalytic site of the rRNA *N*-glycosylase domain of Md1RIP are highly conserved when compared to ricin (Figure 1B panel a).

The type 1 RIP corresponding to gene A (MDP0000918923) was selected for protein expression and characterization of the protein. The transcript for the MdRIP1 gene A encodes a 301 amino acid polypeptide.

The apple type 2 RIP (Md2RIP) gene (MDP0000711911) resembles the classical type 2 RIP genes found in other plant species and shares the highest sequence identity to SNA-I (48% sequence identity and 62% sequence similarity). The transcript for the MdRIP2 gene yielded a deduced amino acid sequence corresponding to a 22 amino acid residue signal peptide (Figure S3 in the Supplementary Materials) followed by a 526 amino acid polypeptide covering the RIP domain and the lectin domain. The sequence contains seven putative *N*-glycosylation sites, four in the RIP domain, and three in the lectin domain. Compared to ricin, most amino acid residues that compose the catalytic site are conserved in the Md2RIP sequence (Figure S2 in the Supplementary Materials). Sequence alignment with ricin revealed a single amino acid substitution in the active site (Y80 of ricin is replaced by S71). Alignment with SNA-I further indicates that all four intra-chain disulphide-bridges that stabilize the lectin domain (C24–C43, C65–C77, C147–C162, C198–C205) are conserved in the apple RIP (Figure S3 in the Supplementary Materials). Furthermore, the amino acids that built the two carbohydrate binding domains are highly conserved between Md2RIP and SNA-I and ricin (Figure S2 in the Supplementary Materials) [20,21,22].

### 2.3. Purification and Characterization of Recombinant Md1RIP and Md2RIP

**Figure 2:**
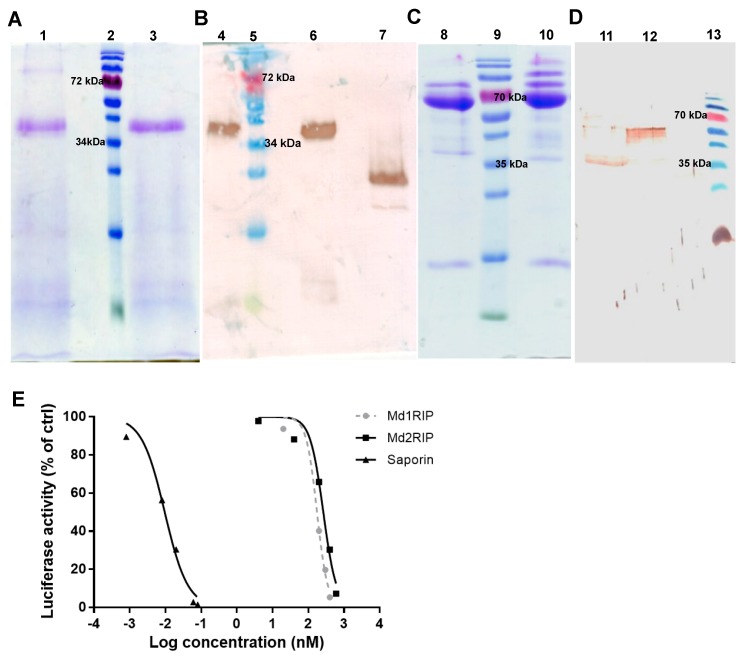
SDS-PAGE (**A**,**C**) and Western blot analysis (**B**,**D**) of recombinant MdRIPs. Lanes 1 and 4: unreduced Md1RIP, lanes 3 and lane 6: reduced Md1RIP treated with β-mercaptoethanol, lane 7: positive control (Orysata, [23]), lanes 8 and 12: reduced Md2RIP, lane 10: unreduced Md2RIP, lane 11: positive control (Md1RIP). In panels A and B 5 µg protein was loaded in each well. In panels C and D 15 µg and 3 µg protein were loaded, respectively. Protein ladder (Fermentas) was run in lanes 2, 5, 9, and 13; (**E**) Effect of RIPs in a cell-free translation assay. Dose response curves for luciferase synthesis were measured after treatment with increasing concentrations of Md1RIP, Md2RIP, and saporin for 30 min. Luciferase activity is shown as a function of the concentration of Md1RIP and Md2RIP.

Heterologous expression of the MdRIP sequences allowed the purification of recombinant Md1RIP and Md2RIP from *Pichia pastoris* and *Nicotiana tabacum* cv. Bright Yellow-2 (BY-2) cells, respectively. SDS-PAGE analysis revealed that the molecular mass of the recombinant Md1RIP polypeptides was around 40 kDa (Figure 2A), which was approximately 4 kDa higher than the calculated molecular mass of the Md1RIP sequence including the c-myc and (His)_6_ tags (36.1 kDa) (Figure S4C in the Supplementary Materials). Western blot analysis with a monoclonal antibody directed towards the His tag (Figure 2B) confirmed that the 40 kDa band corresponded to the recombinant MdRIP. Furthermore, Edman degradation of the recombinant Md1RIP yielded the sequence EAEAEFALSFSI (Figure S4 in the Supplementary Materials). Since the EA repeats in this sequence correspond to part of the α-mating sequence it can be concluded that the signal peptide sequence included in the construct to achieve secretion of the recombinant protein was not completely cleaved by the Ste13 protease.

SDS-PAGE analysis (Figure 2C) of recombinant Md2RIP yielded a major polypeptide band of 65.5 kDa. The size of these polypeptides is approximately 4 kDa higher than the calculated molecular mass of the Md2RIP coding sequence (61.5 kDa, RIP and lectin domain containing a (His)_6_ tag). Western blot analysis using a polyclonal antibody against the Md1RIP confirmed that the 65.5 kDa polypeptide reacts with the RIP antibody (Figure 2D). Edman degradation of the 65.5 kDa polypeptide yielded the sequence of GATAXXDIXXL (Figure S4 in the Supplementary Materials), which suggested that, besides the signal peptide, an extra propeptide of 27 amino acid residues is cleaved at the *N*-terminus of the recombinant Md2RIP secreted to the BY-2 cell medium.

The slightly higher mass of the recombinant polypeptides for both Md1RIP and Md2RIP compared to the calculated mass of the RIP sequences was due to *N*-glycosylation of the recombinant proteins, as shown by *N*-glycosidase treatment of the recombinant proteins and subsequent SDS-PAGE analyses (Figure S5 in the Supplementary Materials).

### 2.4. Biological Activities of Recombinant MdRIPs

To assess the catalytic activity of the MdRIPs, a cell-free translation system was used to study the inhibition of protein synthesis by recombinant MdRIPs. In a parallel experiment saporin, the type 1 RIP from *Saponaria officinalis* was analyzed as a control. As shown in Figure 2E, all RIPs tested showed a clear reduction of protein synthesis. Higher inhibition of protein translation was observed with increasing concentrations of the RIPs, suggesting a concentration-dependent protein activity. The 50% inhibitory concentration (IC_50_) for Md1RIP and Md2RIP corresponded to 175 nM and 263 nM, compared to an IC_50_ value of 9.6 pM for saporin.

The carbohydrate binding activity of the recombinant Md2RIP was analyzed by agglutination assays as well as glycan array screening. The recombinant protein behaved as a genuine lectin, the minimal concentration of recombinant protein required for agglutination of trypsin treated rabbit erythrocytes being 1.55 µg/mL. A detailed analysis of the carbohydrate binding specificity using glycan microarray screening revealed that the Md2RIP reacts preferentially with Neu5Ac (glycan #11) and glycans carrying at least one terminal Neu5Ac residue as well as with 2-keto-3-deoxy-d-glycero-d-galactononic acid (KDN)α2-6Galβ1-4GlcNAc (glycan #357) (Figure S6 and Table S2 in the Supplementary Materials). Furthermore, the Md2RIP interacted also with Neu5Gc (glycan #286) but less strongly than with Neu5Ac.

### 2.5. Molecular Modeling of Enzymatically Active Sites and Carbohydrate-Binding Sites

**Figure 3:**
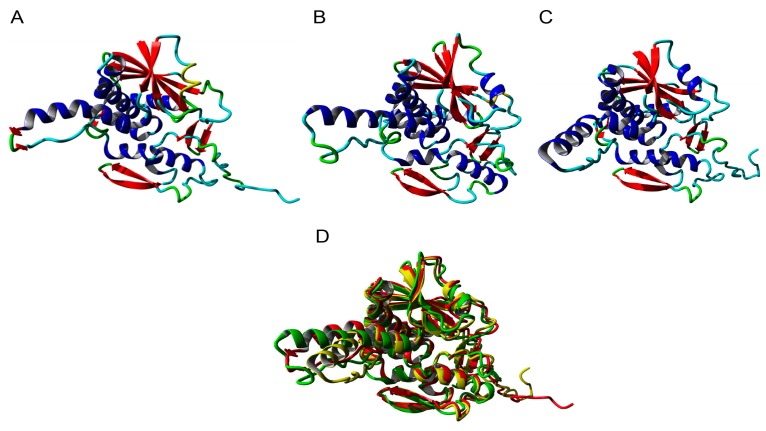

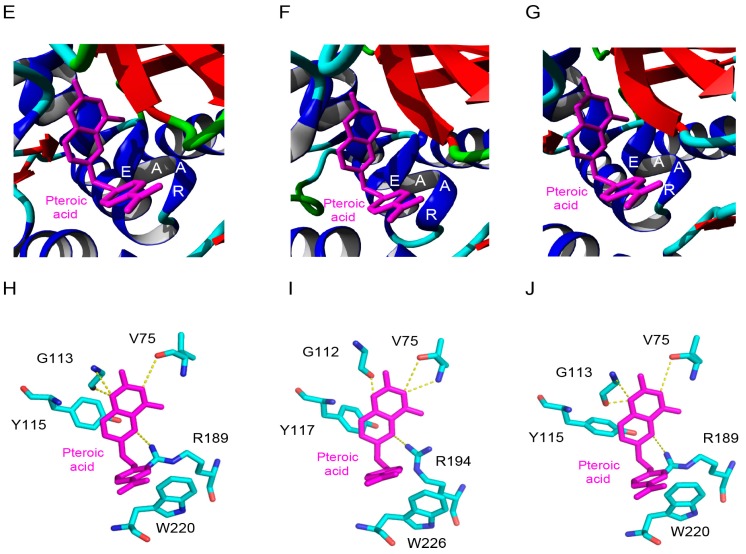
Ribbon diagrams built for the type 1 RIPs from apple (**A**), peach (**B**), and pear (**C**). α-helices, β-sheets and loops/turns, are colored red, blue, and cyan, respectively. (**D**) Superposition of the type 1 RIP models of apple (colored red), peach (colored green), and pear (colored yellow). Docking of pteroic acid (magenta stick) into the active site of type 1 RIPs of apple (**E**), peach (**F**), and pear (**G**), showing the EAAR sequence of the active site. Network of H-bonds (dashed yellow lines) anchoring pteroic acid to the active site in type 1 RIP of apple (**H**), peach (**I**), and pear (**J**). A stacking interaction occurs between pteroic acid and Tyr and Trp residues located in the vicinity of the active site.

Since all type 1 RIPs from Rosaceae share a high sequence similarity, one type 1 RIP sequence for each fully sequenced Rosaceae genome was selected to perform the molecular modelling studies. The modelled type 1 RIPs of apple (*Malus domestica*), peach (*Prunus persica*), and pear (*Pyrus communis*), consist of a single RIP domain sharing the conserved organization of a left-twisted bundle of β-sheets associated to α-helices (Figure 3A–C). They only differ from each other by the shape and the size of the loops and turns connecting the β-sheets and the α-helices. Accordingly, the three models superpose nicely but some discrepancies exist for the loop and turn structures (Figure 3D). All RIPs contain the sequence stretch EAAR involved in the rRNA *N*-glycosylase activity of the RIP domain (Figure 3E–G), and docking experiments performed with pteroic acid suggest they readily accommodate the substrate analog through a typical network of hydrogen bonds and stacking interactions with aromatic residues (Figure 3H–J).

The type 1 RIPs from the Rosaceae species exhibit the canonical RIP-fold, characterized by the occurrence of three domains, namely the *N*-terminal domain 1 made of β-sheets and α-helices, the central domain 2 built up from α-helices, and the C-terminal domain 3 which consists of one or two α-helices followed by two shorts strands of antiparallel-sheet organized in a β-hairpin (Figure S7A–C in the Supplementary Materials). The occurrence of this α-helix-β-hairpin domain 3 in type 1 RIPs is believed to be essential for some of their biological activities, including the binding to a lipid bilayer and the rRNA *N*-glycosylase activity [5,24,25]. Although Rosaceae type 1 RIPs possess the α-helix-β-hairpin domain at the C-terminal end of the polypeptide chain (Figure S7D–F in the Supplementary Materials) the degree of conservation of this β-hairpin domain is rather weak, compared to other structural domains 1 and 2, which exhibit a higher degree of conservation (Figure S7G–I in the Supplementary Materials). The α-helix-β-hairpin in the RIP domain of apple, peach, and pear shows an amphipathic conformation with opposing nonpolar and polar faces similar to BE27 (Figure S8 in the Supplementary Materials) [26].

**Figure 4:**
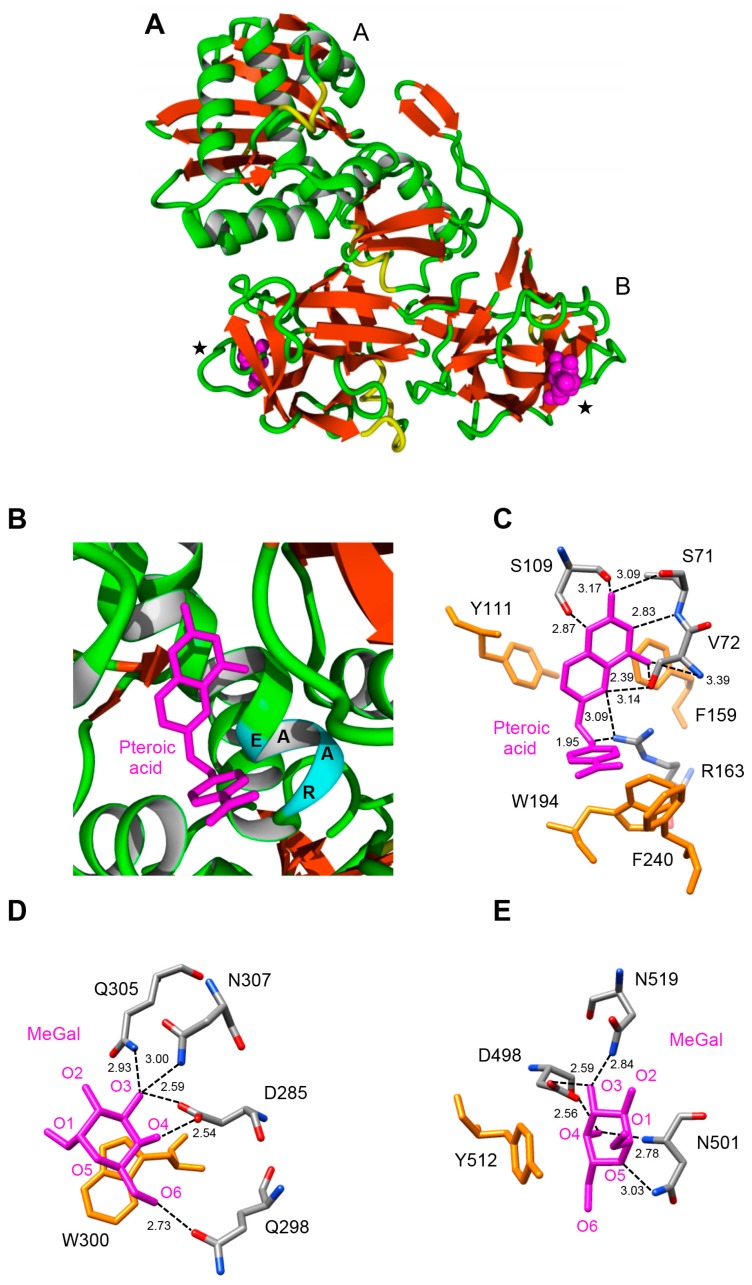
Molecular organization of Md2RIP. (**A**) Ribbon diagram showing the organization of Md2RIP with a RIP (A) and a lectin (B) domain. Stars show the position of the carbohydrate-binding sites in the lectin domain; (**B**) Docking of pteroic acid (magenta stick) into the active site of the RIP domain, showing the EAAR sequence of the active site; (**C**) Network of H-bonds (dashed lines) anchoring pteroic acid to the active site of the RIP domain. H-bond distances are expressed in angström (Å). Aromatic residues developing a stacking interaction with pteroic acid are colored orange; (**D**,**E**) Network of hydrogen bonds (dashed lines) anchoring MeGal (magenta stick) to the amino acid residues (atom-code colored sticks) forming the carbohydrate-binding sites 1 (**D**) and 2 (**E**) of Md2RIP lectin domain. Aromatic residues interacting through a stacking with the pyranose ring of MeGal in both sites are colored orange.

The RIP domain of MdRIP2 exhibits an overall three-dimensional fold similar to that found in other type 2 RIPs, built up of six strands of β-sheet clustered in a left-handed twisted bundle, associated to eight α-helices (Figure 4A). The lectin domain consists of two tandemly arrayed domains 1 and 2, each consisting of four subdomains (1λ, 1α, 1β, and 1γ for domain 1; 2λ, 2α, 2β, and 2γ for domain 2), in which strands of β-sheet predominate. The RIP domain contains the sequence stretch 160EAAR163, which plays a key role in the rRNA *N*-glycosylase activity of the RIP domain (Figure 4B). The key residues of the active site of the RIP domain (S71, V72, S109, Y111, R163), readily accommodate pteroic acid as shown from docking experiments (Figure 4C). The hydrogen bond network anchoring pteroic acid to the active site of the RIP domain within Md2RIP resembles that observed in the ebulin-A-pteroic acid complex [27]. Additional stacking interactions occur with the aromatic residues Y111, F159, W194, and F240.

The lectin domain of MdRIP2 contains two carbohydrate-binding sites occurring at both ends of subdomains 1α (D285, Q298, W300, Q305, N307) and 2γ (D498, N501, Y512, N519) (Figure 4D,E). Similar to the ricin-B chain, the carbohydrate-binding site of subdomain 2γ of Md2RIP differs from that observed in the ebulin-B chain by the replacement of a Phe residue (F) by Y250. However, these aromatic residues create a stacking interaction with the pyranose ring of galactose that reinforces the interaction with the sugar. Both sugar-binding residues readily interacted with methyl-α-d-galactopyranoside (MeGal) in docking experiments (Figure 4E,F) through a network of hydrogen bonds similar to that observed in the galactose-ebulin-B complex [28], but rather different from that observed in ricin [29]. Similar to the ebulin-galactose complex, an additional residue participates in the network of hydrogen bonds anchoring the sugar to both carbohydrate-binding sites. Obviously, these additional interactions reinforce the binding of galactose to the lectin moiety.

**Figure 5:**
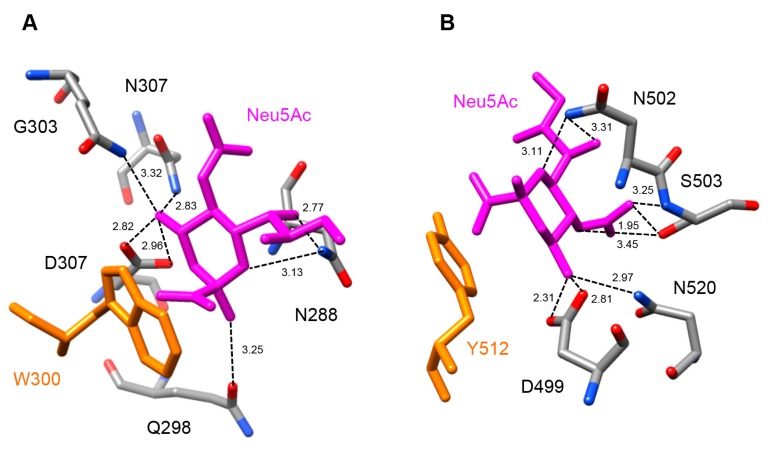
Docking of Neu5Ac to the carbohydrate-binding sites of Md2RIP. (**A**) Network of hydrogen bonds (dashed lines) anchoring Neu5Ac to the carbohydrate-binding site of subdomain 1α of Md2RIP. H-bond distances are expressed in angström (Å). The W300 aromatic residue (colored orange) participates in a stacking interaction with the pyranose ring of Neu5Ac; (**B**) Network of hydrogen bonds (dashed lines) anchoring Neu5Ac to the carbohydrate-binding site of subdomain 1γ of Md2RIP. The Y512 aromatic residue (colored orange) participates in a stacking interaction with the pyranose ring of Neu5Ac.

Docking experiments performed with sialic acid Neu5Ac suggest that both lectin domains of Md2RIP readily accommodate the sugar derivative (Figure 5A,B). Sialic acid anchors to both binding sites through a network of seven and eight hydrogen bonds, respectively. An additional stacking interaction with an aromatic residue, W300 in site 1 and Y512 in site 2, should reinforce the interaction of Neu5Ac with the lectin. A three-dimensional model with similar fold and binding properties towards pteroic acid (RIP domain) and MeGal/Neu5Ac (lectin domain), was built by homology modeling of the type 2 RIP sequence from *Pyrus communis* (PCP031611) (results not shown).

## 3. Discussion

Ribosome-inactivating proteins are widely distributed in flowering plants. In this study, the genomes of Rosaceae species were screened for RIP sequences. Evidence for the occurrence of multiple RIP sequences was obtained for the genomes of *Malus domestica*, *Prunus persica*, *Prunus mume*, *Pyrus communis*, and *Pyrus bretschneideri* but RIP sequences were absent from the genomes of *Fragaria vesca* and *Fragaria ananassa*. The latter result is in contradiction to a recent report by Polito et al. [30] who reported increased RIP activity in strawberry (*Fragaria ananassa*) plants grown under reproductive, biotic, and drought stress conditions. However, since only partially purified basic protein fractions from strawberry tissue extracts were used to perform the RIP assays, the RIP activity reported in this study is questionable. Previously Di Maro et al. reported that the *Malus domestica* genome encodes four type 1 RIPs [5]. However, for one of them the open reading frame is interrupted and the sequence is incomplete. In this study we focused on the three fully sequenced Md1RIP genes.

Both type 1 and type 2 RIP sequences have been retrieved from Rosaceae species. The genomes of apple (*Malus domestica*) and pear (*Pyrus communis*) express both type 1 and type 2 RIPs, whereas peach (*Prunus persica* and *Prunus mume*) and pear (*Pyrus bretschneideri*) express a complex set of type 1 RIPs. At present, only a few species (*Sambucus ebulus*, *Iris* x *hollandica, Momordica charantia*, and *Trichosanthes kirilowii*) have been reported to contain both type 1 and type 2 RIPs [11,13,14,15,16,31,32]. These species present interesting models to study the evolutionary relationships between type 1 and type 2 RIPs.

Phylogenetic analysis suggested that most type 1 RIPs found in dicots are evolutionary related to type 2 RIPs [6]. The type 1 RIPs from *Iris* x *hollandica* are derived from the deletion of the lectin domain of the type 2 RIPs. Similarly, the type 1 RIP genes from Rosaceae lack the signal peptide as well as the lectin domain of the type 2 RIP sequence. Therefore, type 1 RIPs from Rosaceae can be considered as “deletion” products of type 2 RIPs [5]. The type 2 RIP gave rise to the multiple lines of type 1 RIP genes (e.g., Md1RIP-A, B, or C) by lectin domain deletion/gene truncation events [18]. Another striking difference between type 1 and type 2 RIP sequences from apple/pear involves the lack of a signal peptide in the type 1 RIP sequences. Consequently, the type 1 RIPs are likely to be synthesized on free ribosomes in the cytoplasm whereas type 2 RIPs will follow the secretory route for protein synthesis. The different location for type 1 and type 2 RIPs in the plant cell can be important for their particular role in the plant.

The recombinant Md1RIP secreted by the *P. pastoris* cells is a functional protein, although the processing of the α-mating sequence from *S. cerevisiae* was not correctly cleaved by the Ste13 protease. Problems with the correct processing of this α-mating sequence have been reported before on several occasions [23]. The recombinant Md1RIP was made as a secreted glycosylated protein. Since the native Md1RIP produced by the apple cells is synthesized without a signal peptide, it does not enter the secretory pathway and hence it is unlikely that apple Md1RIP occurs as a glycoprotein. Despite the incorrect/incomplete processing of the *N*-terminal α-mating sequence, the presence of a His tag at the C-terminus, and the presence of *N*-glycans the recombinant Md1RIP is catalytically active.

Tobacco BY-2 cells were selected for the production of the recombinant Md2RIP since transgenic tobacco plants and cells are fully capable of carrying out the cleavage of the type 2 RIP precursors and glycosylation of the protein [33]. Indeed, BY-2 cells successfully expressed and secreted Md2RIP into the BY-2 medium, the final concentration exceeding 10 mg per liter. Characterization of Md2RIP revealed a unique protein structure. The results from SDS-PAGE and western blot analysis indicate that the RIP and lectin domain are located on a single polypeptide in the recombinant Md2RIP, which implies that the processing step reported for type 2 RIPs whereby the linker between the RIP and lectin chain is excised from the precursor protein does not take place for the Md2RIP. Furthermore, preliminary data confirmed the lack of processing of the Md2RIP precursor protein in apple. Western blot analysis of a crude extract from immature (10 days post-pollination) apple (*M. domestica* cv. ‘Golden Delicious’) fruits yielded a polypeptide with a molecular mass of approximately 66 kDa, corresponding to a polypeptide that contains both the RIP and lectin domain.

The results of in silico analysis and molecular modelling revealed that the amino acids known to be important for the activity of ricin are highly conserved in the active site of the MdRIP sequences, suggesting that the apple RIPs are functional. Furthermore, translation inhibition experiments confirmed that the recombinant type 1 and type 2 RIPs from apple inhibit protein synthesis. However, the catalytic activity of Md1RIP (IC_50_ = 175 nM) and Md2RIP (IC_50_ = 263 nM) is much lower (at 10^4^ level) than that of saporin, 30–50 folder lower than SNA-I [34] and four orders of magnitude inferior to the values reported for ricin (IC_50_ = 100 pM, [35]). According to previous work [36] the replacement of Y80 of ricin by S71 accounts for a 160-fold reduction in the catalytic activity. Hence, this amino acid substitution in the Md2RIP sequence can contribute to the higher IC_50_ value. However this reasoning does not hold true for the Md1RIP in which the Y80 of ricin is conserved. Therefore, it is likely that residues other than those involved in the canonical active site also influence the catalytic properties of the MdRIPs. It has been reported that glycosylation can affect RIP activity. Protein synthesis inhibition activity of the recombinant PD-L1 (a type 1 RIP from *P. dioica*) was increased after removal of the glycan chains [9]. In this study, the recombinant Md1RIP was synthesized following the secretory pathway for protein synthesis, though the native protein is most probably synthesized in the cytoplasm due to the lack of a signal peptide in the sequence. The deduced sequence of Md1RIP comprises five putative *N*-glycosylation sites (Figure S4 in the Supplementary Materials) and our data show that the recombinant protein indeed contains some *N*-glycan chains. It is possible that these glycans affect the enzymatic activity of the recombinant Md1RIP.

The Md2RIP behaves as a genuine lectin with a well-defined carbohydrate binding activity and specificity. Sequence alignment results indicated that S197 of SNA-I, which is critical for the binding to sialic acid in the Neu5Ac(α2-6)Gal/GalNAc sequence of 2-6-sialyllactose, is conserved the type 2 RIP sequences from *M. domestica* and *P. communis* (Figure S3 in the Supplementary Materials). Furthermore, a detailed glycan microarray analysis revealed that the recombinant Md2RIP preferentially interacts with Neu5Ac and glycans carrying at least one terminal Neu5Ac(α2-6)Gal/GalNAc residue, and also strongly binds to KDN. In addition, the Md2RIP reacted with a non-sialylated complex glycan. Though the preferential interaction with Neu5Ac and glycans carrying terminal Neu5Ac(α2-6)Gal/GalNAc residue is reminiscent of the carbohydrate binding specificity of SNA-I the results summarized in Table S2 in the Supplementary Materials leave no doubt that there are major differences between both lectins. For example, the Md2RIP has a much higher affinity for Neu5Ac than for Neu5Ac(α2-6)Galβ1-4GlcNAc whereas for SNA-I this is the reverse. Though still speculative, the latter observation might indicate that the binding site of the Md2RIP is less extended compared to that of SNA-I. The carbohydrate binding properties of the apple RIP are similar to those of SNA-I. However, this carbohydrate binding specificity is very different from the majority of type 2 RIPs that specifically react with galactose or galactose derivatives. Although it is commonly accepted that sialic acid is absent in plants [37], sialic acid is widely spread from bacteria to animal tissues, and plays an important role in cell communication, adhesion, and protein targeting [38]. Similar to SNA-I, the sialic acid binding specificity of the apple type 2 RIP may also protect plants from plant diseases e.g., fungi, insects, or viruses [39]. Recently Hamshou et al. [40] reported strong aphicidal activity of the RIPs from apple when tested in an artificial diet and in planta using transgenic tobacco lines overexpressing the RIPs.

Interestingly, the Md1RIP sequence possesses an α-helix-β-hairpin structure at the C-terminus. This structural motif is also present in other Rosaceae type 1 RIPs (*Pyrus communis* and *Prunus persica*). Citores et al. [25] reported the antifungal activity of BE27, a type 1 RIP from *Beta vulgaris* L., against the green mould *Penicillium digitatum*. They hypothesize this C-terminal α-helix-β-hairpin motif can assist BE27 insertion into the lipid bilayer of fungal membranes and inactivate the fungal ribosomes. Our data show that the α-helix-β-hairpin motif is also present in the cytoplasmic type 1 RIPs from Rosaceae species. It remains to be shown that this motif can help to protect the plant against fungal infection.

Our study indicates that most genomes from Rosaceae encode one or more RIP sequences. Sequence comparisons and molecular modelling studies leave no doubt that all these Rosaceae RIPs are highly similar with conservation of the amino acids important for the catalytic activity of the RIP domain and carbohydrate binding activity of the lectin domain. However, our data do not allow any conclusions with respect to the toxicity of the RIPs. According to EST data available, RIPs are expressed in young tissues and unripe fruits in low amounts. At present there is no evidence for RIP expression in the mature fruits. Since the fruits are widely consumed in large quantities it is unlikely that the RIPs are toxic, but they could exert beneficial antiviral and insecticidal activity [40] or could cause allergy in atopic individuals, as some RIPs do [41]. Our data can contribute to the understanding of evolution of RIP genes in Rosaceae and will help to deduce their biological role. Future work will focus on the expression patterns of RIPs in different plant tissues and the physiological importance of these proteins.

## 4. Materials and Methods

### 4.1. Sequence Alignment and Phylogenetic Analysis

Type 1 RIP (trichosanthin, accession number AAB31048.1) and type 2 RIP (cinnamomin, AAK82460.1; Ebulin, AJ400822; ricin, XP_002534649.1; SNA-I, U27122) sequences were used as queries to search for RIP sequences in NCBI database (*Pyrus bretschneideri* and *Prunus mume*), Genome database for Rosaceae (*Malus domestica*, *Pyrus communis, Prunus persica*, *Fragaria ananassa* and *Fragaria vesca*), and Phytozome database (*Prunus persica*). Sequence alignments were represented by sequence logos as created by WebLogo 3 [42]. ClustalW was used for the alignment of the RIP sequences. A phylogenetic tree of all RIP sequences was constructed using the constraint-based alignment tool-COBALT [43].

### 4.2. Purification of Recombinant Md1RIP

The sequence for Md1RIP was expressed in *Pichia pastoris*. Therefore, an expression vector containing the coding sequence for Md1RIP was constructed according to EasySelect Pichia Expression Kit from Invitrogen (Invitrogen, Carlsbad, CA, USA). To allow secretion of Md1RIP into the yeast culture medium, the Md1RIP construct contained an α-mating sequence from *Saccharomyces cerevisiae*, upstream of the RIP coding sequence. Furthermore, a polyhistidine tag was provided downstream of the RIP coding sequence for easy purification of the fusion protein. The recombinant Md1RIP was purified using a combination of ion exchange chromatography on S Fast Flow (GE Healthcare, Uppsala, Sweden) and affinity chromatography on a nickel Sepharose column, as described by Desmyter et al. [44] and Al Atalah et al. [23], respectively.

### 4.3. Purification of Recombinant Md2RIP

Bright yellow-2 tobacco cells (BY-2 cells) were used for the expression of Md2RIP. A binary expression vector containing the Md2RIP sequence including the signal peptide of Md2RIP was constructed according to the Gateway™ cloning technology of Invitrogen. Stable transformation of BY-2 cells was performed as reported previously [45]. The recombinant Md2RIP was expressed in BY-2 cells under the control of the 35S Cauliflower Mosaic virus promoter and purified from the BY-2 cell culture medium using hydrophobic interaction chromatography on phenyl Sepharose combined with affinity chromatography on fetuin-Sepharose 4B [21,46].

### 4.4. Western Blot Analysis

Samples were separated by SDS-PAGE and proteins transferred onto a PVDF membrane (Bio Trace™ PVDF, PALL, Gelman Laboratory, Ann Arbor, MI, USA). First, the blots were blocked in blocking buffer, consisting of 5% milk powder dissolved in Tris buffered saline (TBS: 10 mM Tris, 150 mM NaCl and 0.1% (*v*/*v*) Triton X-100, pH 7.6). After blocking, blots were incubated for 1 h in TBS supplemented with the following primary antibodies: (i) for recombinant Md1RIP purified from *Pichia pastoris* medium: mouse monoclonal anti-His (C-terminal) antibody (1:5000, Invitrogen; (ii) for recombinant Md2RIP purified from tobacco cells: rabbit polyclonal anti-type 1 RIP antiserum (1:1500, produced by Thermo Scientific by injecting two rabbits with recombinant type 1 RIP from apple). The secondary antibody was a 1:1000 diluted rabbit anti-mouse IgG (Dako Cytomation, Glostrup, Denmark) or a 1:5000 diluted horseradish peroxidase-coupled goat anti-rabbit IgG (Sigma-Aldrich, St. Louis, MO, USA), respectively. Since Md1RIP shares 43% sequence identity with the RIP domain of Md2RIP the antiserum raised against Md1RIP also recognized the recombinant Md2RIP. Immunodetection was achieved by a colorimetric assay using 3,3′-diaminobenzidine tetrahydrochloride (Sigma-Aldrich) as a substrate. All washes and incubations were conducted at room temperature with gentle shaking.

### 4.5. N-terminal Sequence Analysis

Purified Md1RIP and Md2RIP were analyzed by SDS-PAGE, electroblotted onto a Problot™ polyvinylidene fluoride (PVDF) membrane (Applied Biosystems, Foster City, CA, USA) and the blot stained using 1:1 mix of Coomassie Brilliant Blue and methanol to visualize the protein. The bands of interest were cut and used for *N*-terminal sequencing by Edman degradation using a capillary Procise 491cLC protein sequencer without alkylation of cysteines (Applied Biosystems).

### 4.6. Biochemical Assays

A cell-free system, the TnT^®^ T7 Quick Coupled Transcription/Translation System Kit (Promega, Mannheim, Germany) was used to determine and quantify the protein synthesis inhibition activity of recombinant Md1RIP and Md2RIP as described by Shang et al. [34]. The prepared mixture was incubated at 30 °C for 10 min and chilled on ice. Subsequently, 2 µL PBS or PBS containing different concentrations of proteins were added to the reaction mixture and incubated for 30 min at 30 °C. After addition of 35 µL nuclease-free water at room temperature, the reaction samples were transferred to a luminometer plate (Greiner Labortechnik, Frickenhausen, Germany) containing 5 µL luciferase assay reagent at 25 °C. The relative luciferase activities of the samples were determined at 562 nm for 10 s using a microtiter top plate reader (Infinite 200, Tecan, Mannedorf, Switzerland) with an initial delay of 2 s.

Agglutination assays using rabbit erythrocytes (BioMérieux, Marcy I’Etoile, France) were performed in small glass tubes by mixing 10 µL purified recombinant Md2RIP, 10 µL of 1 M ammonium sulphate and 30 µL of a 10% suspension of trypsin-treated rabbit erythrocytes. After 30 min at room temperature, the agglutination was assessed visually.

Glycan microarrays were printed as described previously [47]. The printed glycan array contains a library of natural and synthetic glycan sequences representing major glycan structures of glycoproteins and glycolipids. Array version 5.0 with 611 glycan targets, was used for the analyses with recombinant Md2RIP [48].

### 4.7. Protein Deglycosylation

Recombinant MdRIPs were digested with *N*-glycosidase F (PNGase F) as described by Al Atalah et al. [49]. Briefly, 2 µg of recombinant proteins were mixed in a volume of 10 µL denaturation buffer (0.5% SDS and 0.04 M dithiothreitol). The samples were boiled at 100 °C for 10 min and cooled down to room temperature. To the denatured samples we added: 2 µL of 10× reaction buffer (0.5 M sodium phosphate pH 7.5), 2 µL 10% NP-40, and 5.5 µL distilled water to reach a total volume of 20 µL. The samples were incubated at 37 °C for 4 h after adding 0.5 µL of PNGase F (1000 U·µL^−1^) to each sample. RNAse B (2 µg) was used as a positive control. Finally, protein samples were analyzed by SDS-PAGE.

### 4.8. Molecular Modeling and Docking

Homology modeling of type 1 RIPs from apple (*Malus domestica*, type 1 RIP GDR accession number MDP0000918923; type 2 RIP MDP0000711911), peach (*Prunus persica*, ppa009409mg), and pear (*Pyrus communis*, type 1 RIP-PCP001408.1; type 2 RIP accession no. PCP031611), was performed with the YASARA Structure program [50], running on a 2.53 GHz Intel core duo Macintosh computer. Different models of type 1 RIPs were built from the X-ray coordinates of ricin A-chain (PDB codes 2PJO, 2VC3, 1IFT) [51,52,53] and mutant N122a (PDB code 1UQ5) and R213d (1UQ4) of recombinant ricin A-chain [28], used as templates. Finally, a hybrid model for the three type 1 RIPs was built up from previous models. Different models of type 2 RIP from apple and pear, were similarly built using the X-ray coordinates of the snake gourd seed lectin (PSB code 4HR6) [54], ebulin (PDB code 1HWM) [27], native mistletoe lectin ML-I (PDB codes 2RG9, 1ONK), and ML-1 complexed to GlcNAc (PDB code 4EB2), as templates. A hybrid model of the two type 2 RIPs was finally built from the previous models. PROCHECK [55], ANOLEA [56], and the calculated QMEAN scores [57,58], were used to assess the geometric and thermodynamic qualities of the three-dimensional models (Table S3). Using ANOLEA to evaluate the models, only a few residues of the type 1 RIP and type 2 RIP models exhibited an energy over the threshold value. Both residues are mainly located in the loop regions connecting the β-sheets to the α-helices in the models. The calculated QMEAN6 score of all of the models gave values > 0.5.

The ConSurf server was used to discriminate between conserved and variable regions in the RIP models [59]. Molecular cartoons were drawn with YASARA [50] and Chimera [60].

## Supplementary Materials

Supplementary materials can be accessed at: http://www.mdpi.com/1420-3049/21/8/1105/s1.
