# Supplementary material for: Ribosome Inactivating Proteins from Rosaceae
Source: Molecules. 2016 Aug 22;21(8):1105. doi: 10.3390/molecules21081105 (PMC6274481; doi:10.3390/molecules21081105)
Supplement: Supplementary file 1 [file molecules-21-01105-s001.pdf]

Supplementary Materials: Ribosome Inactivating Proteins from Rosaceae

Chenjing Shang, Pierre Rougé and Els J.M. Van Damme

Type 1 RIPs

1. *Malus domestica* (MDP0000918923)

MALSFSIKNATTTTYRTFIEALRAQLTAGGSTSHGIPVLRRRQDVKDDQRFVLVNLTNYDSYTITVA
IDVVNAYVVGYCAGTRSYFLRDPATHPPPLHRLFPGTTRTTLPFAGDYLGLGRAAQEALQQNTNRNR
AAGSRIHENISMRERIPLGPGELDNAISQLRYAESASSQAAAFIVIIQIVSEAARFRYIQGQVRDRI
RDGTSAEPDPAMLSLENSWSNLSEQIQMVPANQLLFINNGSVQIRKADNSIVLVKSVDSDAVRGVAF
LLYCGGNPPAPNSESARTSKVTVQKPTLAKKKK

2. *Malus domestica* (MDP0000223290)

MSIPFTLIDATPDSYSRFIDQLRARLTFGTTSQGIRVLPPSRQVGNNARFIYVDLTNYDGVTVTIGI
DVVNAYVMGYEQGEQNYPLQTLPDDPAPVELLFPNTRSAGELPFTGHYASLGEYARRMQNEQPNRRD
QQALNRLSNPMRQNIGLGPSSLHSAIDMLERAATPLSQAGAILVIIQMVSEAARYPYIERQVRESIQ
TGNSFLPDPRMLSLENNWSNLSRQIMGATRAGRESFSTSVSLDDAYQSHGAPPLVVNSVRDSFIQDM
EIALLLHDRGDDRGTDQGNDPENCTPGPSGSGIGRRGXKKPRRQHE

3. *Malus domestica* (MDP0000134012)

MSISFTLIGATPDSYSTFINQLRDRLTFGTTSQGIPVLPSSRQVGNNDRFIYVNLTNYDGVTVTIGI
DVVNVYVMGYEQGGQNYLLGGTLPDEAATVFPNTRAAGELPFRADYGSLGQYARGMPNEQPNRRDQQ
SVNRLRNDMRENIALGPSSLHWAIHMLVHAATSSQASAIIVIIQMVSEAARYPYIERRVRESIQTAN
SFIPDPRMLTLENHWSTLSRQIMEATRAGRESFSTSVSLVDAYQSHGAPPLVVNSVRDSFVQDMEIA
LLLHDRGDDRGTDQGIDPKNCTAGPSVSGRGKKPHDEL

4. *Prunus mume* (XP_016652174.1)

MALSFSTKNTNPQKYRDFIESLRQRLTAGRPKSHDIPVLPRREDVPDAQLFLLLDLTNSGNNTIRLA
IDVVNAYVVGYAAGGRSYFLKENARDNPPPIHTLFRDTTRMPPLDFDGTYTGLSRAAQEAVKRNIAR
DRARNPAVAGLHKDTPILERIPLGRNELDDAINLLSLAPSQSDQAIGFIVVIQMVCEAARFRFIEGL
LRNSMKDVYDPTIPGPATRSLETHWSDLSEEIQRVPANQTQFQKAVVLHNIRNERVEVRSVDSDVVR
GVAMLLYDQNQNANPGPSAKKPLLKNQKPHIGKPTK

5. *Prunus mume* (XP_016652175.1)

MILSFSTKNATPETYRGFIQALRDQLTAGRPTSHGIPVLPRREDVPDAQRFLLVDLTNSQGNTIRLA
IDVVNAYVVGYAADGRAYLLQENARDNRPPIHTLFREATTRIDLGFDGSYSGLSRVAREAVERNTPR
NRARNRAGASAHDNTPVLEQIPMGRNELDTAISLLRSASSPTNQALGFIVIIQMLSEAARFRAIEGL
VRTTMRETYDPLMRGIAMESLETHWSDLSEQIQRAQQRNETGFDRTIVLHNVGNERREVNSVDSPFV
RGVAMLLYDRNGNCNPGSGPHRHDEL

6. *Prunus mume* (XP_008243880.1)

MALVFSTRNATPQTYRTFIDALRLRLTAGRPTSHGIPVLPRKEDVQNAQRFLLVDLTNSENNTITVA
IDVVNAYVVGYAAGGRSYFLAENAPDDKPPIHVLFPGTTRVPTLRFNGTYSGLTRGAVEAVRRRRAG
NRDPNIDEKTPVLEQIFLGRNQLDEAIRLLRSAVSQPEQALGFVVIIQMLSEAARFRQKLRDWSALP

7. *Prunus mume* (XP_008243881)

MALSFSTKNTNPQKYRDFIESLRQRLTAGRPKSHGIPVLPRREDVPDAQRFLLLDLTNSGNNTIRLA
IDVVNAYVVGYAAGGRSYFLKENALDNPPPIHTLFRDTTRMPPLDFDGTYTGLSRAAQEAVKRNIAR
DRARNPAVAGLHKDTPILERIPLGRNELDDAINLLSLAPSQSDQAVGFIVVIQMICEAARFRFIEGL
LRNSMKDVYDPIIPGLAIRSLETHWSDLSEEIQRVPANQTQFQKAVVLHNIRNERVEVRSVDSDVVR
GVAMLLYDRNQNANPGPSAKKPLLKNLKPHIGKPTK

8. ***Prunus*** **persica (ppa009409mg)**

MILSFSTKNATPETYRDFIQALRDQLTAGRPTSNGIPVLPRREDVPDAQRFLLVDLTNSQGNTIRLA
IDVVNAYVVGYAADGRAYLLQENARDNRPPIHTLFRDATTRIDLGFDGSYSGLSRVAREAVERNTPR
NRARNRAGASAHDNTPVLEQIPMGRNELDTAISLLRSASSPTNQALGFIVIIQMLSEAARFRAIEGL
VRTTMRETYDPLMRGLAMESLETHWSDLSEQIQRAQQRNETGFDRTIVLHNVGNERREVNSVDSPFV
RGVAMLLYDRNGNCNPGSGPHRHDEL

9. ***Prunus persica*** **(ppa009637mg)**

MELSFSTKNTTPQKYRDFIESLRQRLTAGRSKSHGIPVLPRREDVPDAQRFLLVDLTNSGNNTIRLA
IDVVNAYVVGYAAGGRSYFLKENARENPPPIHTLFRDTTRMPPLDFDGSYTGLSRAAQEAVKRNIAR
DRARNPAVAGLHPDTPILERIPLGRNELDDAINLLRLAPSQSDQAVGFIVVIQMICEAASMKDVYDP
TIPGPATRSLENHWSDISKEIQRVPANQTQFQKAVVLHNIKNERVEVRSVDSDVVRGVAMLLYDRNQ
NANPGPSAKKPLLKN

10. ***Pyrus bretschneideri*** **(XP_009374990.1)**

MALALSLLNATPKTYTAFIEALRDRLTAGRPTSHGIAVLPRREDVPDAQRFLYVDLTNYNGDTIRVA
IDVVNVYVVGYRSGNKSYILANNAENPAPTHILFPTAPGAGQSTRTMLPFTGDYPALGAYARRTAQP
SASGARNPGSRIHEDIPMLELIPLGRNELDNAITKLHYAASHSDQAAAFIVIIQMVSEAARYRYIES
QVGNRMGIDNRPYIPDPAMRSLETNWSALSEQIQKVPANGNRFNRPIQLTTVNNRPLEVNSVEADMV
QRRGIAMLLYAR

11. ***Pyrus bretschneideri*** **(XP_009375039.1)**

MALALSLLKATPKTYTAFIEALRARLTAGRPTSHGIPVLPRIKDVPDAQRFLYVDLTNYKGDTIRVA
IDVVNVYVVGYRSGNKSYILANDAKKPAPTHTLFPTALGATQSTRTVLPFTGDYPELGPHARRTAQS
SASGAPGSRIHENIPMLEQIPLGRNELDNAISKLHYAASHSDQAAAFIVIIQMVSEAARFRYIESQV
GTRMGIDNPPYIPDPAMRSLENEWSALSEQIQNVPANGNRFSRSIQLTTVNYRPLVVDSVEADMVQR
RGIVMLLNAS

12. ***Pyrus bretschneideri*** **(XP_009346751.1)**

MALALSLLKETPKTYTAFIEALRARLTAGRPTSHGIPVLPRIKDVPDAQRFLYVDLTNYNGDTIGVA
IDVVNVYAVGYRSGNKSYILANDAKKPAPTHTLFPTALGATQSTRTVLPFTGDYPELGPQARRTAQS
SASGAPGSRIHENIPMLEQIPLGRNELDNAISKLHYAASHSDQAAAFIVIIQMVSEAARFRYIESQV
GTRMGIDNPPYIPDPAMRSLENEWSALSEQVQNVPANGNRFSRSIQLTTVNYRPLVVDSVEADMVQR
RGIVMLLNAS

13. ***Pyrus bretschneideri*** **(XP_009346753.1)**

MGTGAAPKRRRARRAALSYVDLTNYNGDTIRVAIDVVNVYVVGYRSGNKSYILANNAENPAPTHILF
PTAPGAAQSTRTMLPFTGDYPALGAYARRTAQPSASGARNPGSRIHEDIPMLEQIPLGRNELDNAIT
KLHYAASHSDQAAAFIVVIQMVSEAARFRYIESQVGTRMGIDNPPYIPDPAMRSLETNWSALSEQIQ
KVPANGKRFSRPIQLTTVNNRPLKVDSVEADMVQRRGIAMLLYAR

14. ***Pyrus communis*** **(PCP001408.1)**

MALSFSIKNATTTTYRTFIEALRAQLTAGGSTSHGIPVLRRRQDVTDDQRFVLVNLTNYDSYTITVA
IDVVNAYVVGYCAGTRSYFLRDPATHPPPLHRLFPGTTRTTLPFAGDYLGLGRAAQEALQQNTNRNR
AAGSRIHENIPMRERIPLGPGELDNAISQLRYAESASSQAAAFIVIIQIVSEAARFRYIQGQVRDRI
RDGTSAVPDPAMLSLENSWSNLSEQIQMVPANQLLFINNGSVQIRKADNSIVLVKSVDSDAVRGVAF
LLYCGGNPPAPNSESARTSKVTVQKPTLAKKKK

15. ***Pyrus communis*** **(PCP026877.1)**

MALALSLLKATPKTYTAFIEALRARLTAGRPTSHGIPVLPRIKDVPDAQRFLYVDLTNYNGDTIRVA
IDVVNVYVVGYRSGNKSYILANDAKKPAPTHTLFPTALGATQSTRTLLPFTGDYPELGPHARRTAQS
SASGAPGSRIHENIPMLEQIPLGRNELDNAISKLHYAASHSDQAAAFIVIIQIVSEAARFRYIESQV
GTRMGIDNPPYIPDPAMRSLENEWSALSEQIQNVPANGNRFSRSIQLTTVNYRPLVVDSVEADMVQR
RGIVMLLN

16. ***Pyrus communis*** **(PCP011148.1)**

MALALSLLYVTPEKYSAFIEALRARLTDGRPTSHGIPVLPRREDVPDDQRFLFVDLTNYNGDTISVA
IDVVNVYVAGYCSGNKSYILKDNAENRARTQILFPTAPSATQSTPIQLPFTGDYGELGGYARRIAQP
SAARYPGSHSHERIPTLELIPLGRNELDNAITMLHYAASRSDQASSDQAAAFIVIIQMVSEAARFRY
IENQVRTRMEENYCPYIPDPAMRSLENNWSALSEQIQNVPANGSRFNRPIQLTNIRNSPHVVDSVEA
DIVQRRGIAILLYSR

Type 2 RIPs

1. *Malus domestica* (MDP0000711911)

MTRVLAIYITLAFSLFLCGTECNISFSTSGATSNSYNTFIKALRAQLTNGATAIYDIPVLNPSVPDS
QRFLLVDLSNNGNNTITVAIDVVNASVVAYRARAARPYFLADAPDEALDILFNDTRGFFLPFTSNYV
DLEKAAEKSRDKIPLGLTPLHNAITSLWNHESEEAAVSLLVIIQTVFEAARFRVIEQRVRNSISSKA
NFIPDPAMLSLENNWLAISWETQHALNGVFSKSIQLRSTNNNLFLVDSVSSSIMAGVAFLFYNCVTF
PNIIKMPVNVVMGKEIDNEICAVQNRTTHISGLEGLCVDVKNGLDSDGNLVQIWPCGQQRNQKWTFQ
PDETIRSMEKCMTAYSTSSPENYVMIYNCTTAVLEATKWALSTDGTITHRRSGLVLTAHEATRGTTL
TIATNSHSPKQGWRVADDVEPTVTSIIGYNDMCLTANDDKSRVWMEYCVPSKNQQQWALYSEGTIRV
NSDRTLCVTSNGHNSSNVIIILKCELKRGDQRWVFKTDGSILNPNAELVMDVKNSDVYLRQIILYPY
YGTPNQQWLPFF

2. *Pyrus communis* (PCP031611)

MLAIYITLAFSLFLYGTECNISFSTNGATSNSYNKFIKALRAQLTNGATRIYDIPVLNPSVPDSQRF
LLVDLSNNGNNTITVAIDVVNVSVVAYRTRAARSYFLADAPDEALDILFNDTRGFFLPFTSNYIDLE
KAAEKSRDKIPLGLTPLHNAITSLWNHESEEAAASLLVIIQTVFEAARFRVIEQRVRNSISSKANFR
SDHAMLSLENNWLAISWETQHALNGVFSKSIQLRSTNNNLFLVDSVSSSIMAGVAFLFYNCHAVTFP
NIIKMPVNVVMGKEIDNEICAVQNRTTRISGLEGLCIDVKNGLDSDGNLVQIWPCGQQRNQKWTFQP
DETIRSMEKCVTAYSTSSLKNYVMIDNCTTAVPEATKWALSTDGTITHRRSGLVLTAHEATQGTTLT
IATNSHSPRQGWRVGDDVEPTVTSIIGYNDMCLTANDDKSRVWMEYCVPSKNQQQWALYSEGTIRVN
SDRTLCVTSNGHNSSNVIIILKCELRRGDQRWVFKTDGSILNPNAELVMDVKNSDVYLREIILYPYY
GTPNQQWLPFF

Figure S1. Amino acid sequences of type 1 and type 2 RIPs from the Rosaceae species *Malus domestica*, *Prunus mume*, *Prunus persica*, *Pyrus bretschneideri* and *Pyrus communis*. The RIP domain is indicated in black, the signal peptide is indicated in red and the lectin domain is indicated in blue.

```
Gene A   MALSFSIKNATTTTYRTFIEALRAQLTAGGSTSHGIPVLRRRQDVKDDQRFVLVNLTNYD 60
Gene B   MSIPFTLIDATPDSYSRFIDQLRARLTFG-TTSQGIRVLPPSRQVGNNARFIYVDLTNYD 59
Gene C   MSISFTLIGATPDSYSTFINQLRDRLTFG-TTSQGIPVLPSSRQVGNNDRFIYVNLTNYD 59
         *::.*:: .**. :*  **: ** :** * :**:** **   ::* :: **: *:*****

Gene A   SYTITVAIDVVNAYVVGYCAGTRSYFLR-DPATHPPPLHRLFPGTTRT-TLPFAGDYLGL 118
Gene B   GVTVTIGIDVVNAYVMGYEQGEQNYPLQ-TLPDDPAPVELLFPNTRSAGELPFTGHYASL 118
Gene C   GVTVTIGIDVVNVYVMGYEQGGQNYLLGGTLPDEAA---TVFPNTRAAGELPFRADYGSL 116
         . *:*:.*****.**:**  * :.* *    . ...    :**.*  :  *** ..* .*

Gene A   GRAAQEALQQNTNR-NRAAGSRIHENISMRERIPLGPGELDNAISQLRYAESASSQAAAF 177
Gene B   GEYARRMQNEQPNRRDQQALNRLSN--PMRQNIGLGPSSLHSAIDMLERAATPLSQAGAI 176
Gene C   GQYARGMPNEQPNRRDQQSVNRLRN--DMRENIALGPSSLHWAIHMLVHAAT-SSQASAI 173
         *. *:   :::.** :: : .*: :   **:.* ***..*. **  *  * :  ***.*:

Gene A   IVIIQIVSEAARFRYIQGQVRDRIRDGTSAEPDPAMLSLENSWSNLSEQIQMVP-ANQLL 236
Gene B   LVIIQMVSEAARYPYIERQVRESIQTGNSFLPDPRMLSLENNWSNLSRQIMGATRAGRES 236
Gene C   IVIIQMVSEAARYPYIERRVRESIQTANSFIPDPRMLTLENHWSTLSRQIMEATRAGRES 233
         :****:******: **: :**: *: ..*  *** **:*** **.**.**  .. *.:

Gene A   FINNGSVQIRKADN--SIVLVKSVDSDAVRGVAFLLYCGGNPPAPNSESARTSKVTVQKP 294
Gene B   FSTSVSLDDAYQSHGAPPLVVNSVRDSFIQDMEIALLLHDRGDDRGTDQGNDPENCTPGP 296
Gene C   FSTSVSLVDAYQSHGAPPLVVNSVRDSFVQDMEIALLLHDRGDDRGTDQGIDPKNCTAGP 293
         * .. *:     .:  . ::*:** .. ::.: : *   ..    .::..  .:  .  *

Gene A   TLAKKK----K------- 301
Gene B   SGSGIGRRGGKKPRRQHE 314
Gene C   SVSGRG----KKPHDEL- 306
         : :       *
```

Figure S2. Alignment of the deduced amino acid sequences of type 1 RIPs from apple (Md1RIP, A-MDP0000918923, B-MDP0000223290, C-MDP0000134012). "*" Means that the amino acids are identical in all sequences; ":" means conserved amino acid conversions, and "." semi-conserved amino acid substitutions.

```
                                                       1
Ricin           MKPGGNTIVIWMYAVATWLCFGS----TSGWSFTLEDNNIFPKQYPIINFTTAGATVQSY 21
Md2RIP          MT----------RVLAIYITLA----FSLFLC----------GTECNISFSTSGATSNSY 14
Pc2RIP          -------------MLAIYITLA----FSLFLY----------GTECNISFSTNGATSNSY 14
SNA-I           MRLV---------AKLLY--LAVLAICGLGIHGALTHPRVTPPVYPSVSFNLTGA--DTY 19
Ebulin          MRVV--------KAAMLYLHIVVLAIYSVGIQG---------IDYPSVSFNLAGAKSTTY 18
                                 :  :                          :.*.  **   :*

Ricin           TNFIRAVRGRLTTGADVRHEIPVLPNRVGLPINQRFILVELSNHAELSVTLALDVTNAYV 81
Md2RIP          NTFIKALRAQLTNGATAIYDIPVLNPS--VPDSQRFLLVDLSNNGNNTITVAIDVVNASV 72
Pc2RIP          NKFIKALRAQLTNGATRIYDIPVLNPS--VPDSQRFLLVDLSNNGNNTITVAIDVVNVSV 72
SNA-I           EPFLRALQEKVILGNHTAFDLPVLNPESQVSDSNRFVLVPLTNPSGDTVTLAIDVVNLYV 79
Ebulin          RDFLKNLRDRVATGTYEVNGLPVLRRESEVQVKNRFVLVRLTNYNGDTVTSAVDVTNLYL 78
                  *:: :: ::  *      :***     :  .:**:** *:*    ::* *:**.*  :

Ricin           VGYRAGN-SAYFFHPDNQEDAEAITHLFTDVQNRYTFAFGGNYDRLEQLAGNLRENIELG 140
Md2RIP          VAYRARAARPYFLADAP-D--EALDILFNDT-RGFFLPFTSNYVDLEKAAEKSRDKIPLG 128
Pc2RIP          VAYRTRAARSYFLADAP-D--EALDILFNDT-RGFFLPFTSNYIDLEKAAEKSRDKIPLG 128
SNA-I           VAFSSN-GKSYFFSGST-A--VQRDNLFVDT-TQEELNFTGNYTSLERQVGFGRVYIPLG 135
Ebulin          VAFSAN-GNSYFFKDAT-E--LQKSNLFLGT-TQHTLSFTGNYDNLETAAGTRRESIELG 134
                *.: :     **:             **  .     : * .**  **  .   *  * **

Ricin           NGPLEEAISALYYYSTGGTQLPTLARSFIICIQMISEAARFQYIEGEMRTRIRYNRRSAP 200
Md2RIP          LTPLHNAITSLWNHE-----SEEAAVSLLVIIQTVFEAARFRVIEQRVRNSISSKANFIP 183
Pc2RIP          LTPLHNAITSLWNHE-----SEEAAASLLVIIQTVFEAARFRVIEQRVRNSISSKANFRS 183
SNA-I           PKSLDQAISSLRTYTLTAGDTKPLARGLLVVIQMVSEAARFRYIELRIRTSITDASEFTP 195
Ebulin          PNPLDGAITSLWY-------DGGVARSLLVLIQMVPEAARFRYIEQEVRRSLQQLTSFTP 187
                   *. **::*             * .::: ** : *****: ** .:*  :

Ricin           DPSVITLENSWGRLSTAIQESN--QGAFASPIQLQRRNGSKFSVYDVSI--LIPIIALMV 256
Md2RIP          DPAMLSLENNWLAISWETQHAL--NGVFSKSIQLRSTNNNLFLVDSVSS-SIMAGVAFLF 240
Pc2RIP          DHAMLSLENNWLAISWETQHAL--NGVFSKSIQLRSTNNNLFLVDSVSS-SIMAGVAFLF 240
SNA-I           DLLMLSMENNWSSMSSEIQQAQ-PGGIFAGVVQLRDERNNSIEVTNFRRLFELTYIAVLL 254
Ebulin          NALMLSMENNWSSMSLEVQLSGDNVSPFSGTVQLQNYDHTPRLVDNFEELYKITGIAILL 247
                :  ::::**.*  :*   * :    . *:  :**:    .   * ..     :  :*.:.

                                                    1 Subdomain 1α
Ricin           YRCAPPPSSQFSL-------LIRPVVP-------NFNADVCMDPEPIVRIVGRNGLCVDV 16
Md2RIP          YNC--VTFPN---------IIKMPVNVVMGKE---IDNEICAVQNRTTHISGLEGLCVDV 286
Pc2RIP          YNCHAVTFPN---------IIKMPVNVVMGKE---IDNEICAVQNRTTRISGLEGLCIDV 288
SNA-I           YGCAPVTSSSYSNNAIDAQIIKMPVFRGGEYE------KVCSVVEVTRRISGWDGLCVDV 27
Ebulin          FRCVATKTTHN--------AIRMPHVLVGEDNKFNDGETCAIPAPFTRRIVGRDGLCVDV 25
                : *                 :  *                .       :* * :***:**

                                                      Subdomain 1β
Ricin           RDGRFHNGNAIQLWPCKSNTDANQLWTLKRDNTIRSNGKCLTTYGYS-PGVYVMIYDCNT 75
Md2RIP          KNGLDSDGNLVQIWPCGQ--QRNQKWTFQPDETIRSMEKCMTAYSTSSPENYVMIYNCTT 344
Pc2RIP          KNGLDSDGNLVQIWPCGQ--QRNQKWTFQPDETIRSMEKCVTAYSTSSLKNYVMIDNCTT 346
SNA-I           RYGHYIDGNPVQLRPCGN--ECNQLWTFRTDGTIRWLGKCLTAS------SSVMIYDCNT 79
Ebulin          RNGYDTDGTPIQLWPCGT--QRNQQWTFYNDKTIRSMGKCMTANGLN-SGSYIMITDCST 82
                : *   :*. :*: **    : ** **:  * ***   **:*:         :** :*.*

                                              Subdomain 1γ
Ricin           AATDATRWQIWDNGTIINPRSSLVLAATSGNSGTTLTVQTNIYAVSQGWLPTNNTQPFVT 135
Md2RIP          AVLEATKWALSTDGTITHRRSGLVLTAHEATRGTTLTIATNSHSPKQGWRVADDVEPTVT 404
Pc2RIP          AVPEATKWALSTDGTITHRRSGLVLTAHEATQGTTLTIATNSHSPRQGWRVGDDVEPTVT 406
SNA-I           VPPEATKWVVSIDGTITNPHSGLVLTAPQAAEGTALSLENNIHAARQGWTVG-DVEPLVT 138
Ebulin          AAEDATKWEVLIDGSIINPSSGLVMTAPSGASRTTLLLENNIHAASQGWTVSNDVQPIAT 142
                .  :**:* :  :*:* .  *.**::* ..   *:* : .* ::  ***    :.:* .*

                            Subdomain 2α                      Subdomain 2β
Ricin           TIVGLYGLCLQANS--GQVWIEDCSSEKAEQQWALYADGSIRPQQNRDNCLTSDSNIRET 193
Md2RIP          SIIGYNDMCLTANDDKSRVWMEYCVPSKNQQQWALYSEGTIRVNSDRTLCVTSNGHNSSN 464
Pc2RIP          SIIGYNDMCLTANDDKSRVWMEYCVPSKNQQQWALYSEGTIRVNSDRTLCVTSNGHNSSN 466
SNA-I           FIVGYKQMCLRENGENNFVWLEDCVLNRVQQEWALYGDGTIRVNSNRSLCVTSEDHEPSD 198
Ebulin          LIVGYNEMCLQANGENNNVWMEDCDVTSVQQQWALFDDRTIRVNNSRGLCVTSNGYVSKD 202
                 *:*   :**  *     **:* *     :*:***: : :** :..*  *:**:    .

                                                Subdomain 2γ
Ricin           VVKILSCGPASSGQRWMFKNDGTILNLYSGLVLDVRASDPSLKQIILYPLHGDPNQIWLP 253
Md2RIP          VIIILKCELKRGDQRWVFKTDGSILNPNAELVMDVKNSDVYLRQIILYPYYGTPNQQWLP 524
Pc2RIP          VIIILKCELRRGDQRWVFKTDGSILNPNAELVMDVKNSDVYLREIILYPYYGTPNQQWLP 526
SNA-I           LIVILKCEG-SGNQRWVFNTNGTISNPNAKLLMDVAQRDVSLRKIILYRPTGNPNQQWIT 257
Ebulin          LIVIRKCQG-LATQRWFFNSDGSVVNLKSTRVMDVKESDVSLQEVIIFPATGNPNQQWRT 261
                :: * .*    . ***.*:.:*:: *  :  ::**   *  *:::*::   * *** *

Ricin           LF--- 255
Md2RIP          FF--- 526
Pc2RIP          FF--- 528
SNA-I           TTHPA 203
Ebulin          QVPQI 207
```

Figure S3. Sequence alignment of type 2 RIPs from *M. domestica* (Md2RIP) and *P. communis* (Pc2RIP), Ricin, Ebulin and SNA-I. "*" Means that the amino acids are identical in all sequences; ":" means conserved amino acid conversions, and "." semi-conserved amino acid substitutions. The *N*-terminal signal peptide and linker are shown in gray. Amino acid residues known to be important for the carbohydrate binding activity of ricin are shown in green; Residues reported to be critical for the binding to sialic acid in the Neu5Ac(α2-6)Gal/GalNAc sequence of 2-6-sialyllactose (according to [1]) are indicated in blue. The amino acids known to be important for the catalytic activity of the *N*-glycosidase domain of ricin are highlighted in yellow. Cys residues involved in disulfide bridges are shown in red. Putative *N*-glycosylation sites are highlighted in pink. Basic residues for 6S-Gal binding [2] are highlighted in gray. Homologous subdomains (α, β, γ) are indicated by arrows.

(A)

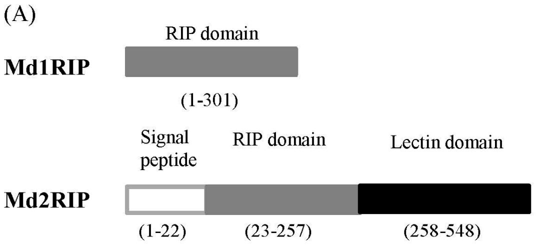

(B)

Md1RIP

M**ALSFSI**KNATTTTYRTFIEALRAQLTAGGSTSHGIPVLRRRQDVKDDQRFVLVNLTNYD 60
SYTITVAIDVVNAYVVGYCAGTRSYFLRDPATHPPPLHRLFPGTTRTTLPFAGDYLGLGR 120
AAQEALQQNTNRNRAAGSRIHENISMRERIPLGPGELDNAISQLRYAESASSQAAAFIVI 180
IQIVSEAARFRYIQGQVRDRIRDGTSAEPDPAMLSLENSWSNLSEQIQMVPANQLLFINN 240
GSVQIRKADNSIVLVKSVDSDAVRGVAFLLYCGGNPPAPNSESARTSKVTVQKPTLAKKK 300
K* 301

Md2RIP

MTRVLAIYITLAFSLFLCGTECNISFSTSGATSNSYNTFIKALRAQLTN**GATA**IY**DI**PV**L** 60
NPSVPDSQRFLLVDLSNNGNNTITVAIDVVNASVVAYRARAARPYFLADAPDEALDILFN 120
DTRGFFLPFTSNYVDLEKAAEKSRDKIPLGLTPLHNAITSLWNHESEEAAVSLLVIIQTV 180
FEAARFRVIEQRVRNSISSKANFIPDPAMLSLENNWLAISWETQHALNGVFSKSIQLRST 240
NNNLFLVDSVSSSIMAGVAFLFYNCVTFPNIIKMPVNVVMGKEIDNEICAVQNRTTHISG 300
LEGLCVDVKNGLDSDGNLVQIWPCGQQRNQKWTFQPDETIRSMEKCMTAYSTSSPENYVM 360
IYNCTTAVLEATKWALSTDGTITHRRSGLVLTAHEATRGTTLTIATNSHSPKQGWRVADD 420
VEPTVTSIIGYNDMCLTANDDKSRVWMEYCVPSKNQQQWALYSEGTIRVNSDRTLCVTSN 480
GHNSSNVIIILKCELKRGDQRWVFKTDGSILNPNAELVMDVKNSDVYLRQIILYPYYGTP 540
NQQWLPFF* 548

(C)

MRFPSIFTAVLFAASSALAAPVNTTTEDETAQIPAEAVIGYSDLEGDFDVAVLPPFSNSTNNGLLFINTTI
ASIAAKEEGVSLEKRE^{86}**A**^{87}**EA**^{89}**EF**ALSFSIKNATTTTYRTFIEALRAQLTAGGSTSHGIPVLRRRQDVKDD
QRFVLVNLTNYDSYTITVAIDVVNAYVVGYCAGTRSYFLRDPATHPPPLHRLFPGTTRTTLPFAGDYLGLG
RAAQEALQQNTNRNRAAGSRIHENISMRERIPLGPGELDNAISQLRYAESASSQAAAFIVIIQIVSEAARF
RYIQGQVRDRIRDGTSAEPDPAMLSLENSWSNLSEQIQMVPANQLLFINNGSVQIRKADNSIVLVKSVDSD
AVRGVAFLLYCGGNPPAPNSESARTSKVTVQKPTLAKKKK***GLQKLISEEDLNSAVDHHHHHH***

Figure S4. Sequence information of RIPs from *Malus domestica* cv. Golden Delicious. (**A**) Schematic diagrams of protein domains in MdRIPs. Md1RIP sequence consists of a RIP domain (amino acids 1-301). Md2RIP consists of a signal peptide (amino acids 1-22) followed by RIP domain (amino acids 23-257) and lectin domain (amino acids 258-548); (**B**) Predicted amino acid sequences of apple Md1RIP (GDR accession no. MDP0000918923) and Md2RIP DNA (GDR accession no. MDP0000711911). The termination codon is marked with an asterisk (*). The signal peptide and the lectin domain within the Md2RIP sequence are highlighted in yellow and green, respectively; (**C**) Deduced sequence of apple type 1 RIP construct expressed in *Pichia*. Note that the apple sequence is preceded by an *N*-terminal signal peptide from yeast (in bold) necessary for secretion and followed by a C-terminal tag containing a c-myc epitope and a $(His)_6$ tag (shown in bold and italic). The cleavage sites for the α-mating factor secretion signal sequence are indicated (Kex2 protease site at position 86 and Ste 13 protease sites at positions 87 and 89). The *N*-terminal sequence of recombinant MdRIPs determined by Edman degradation is underlined. Putative *N*-glycosylation sites are highlighted in gray.

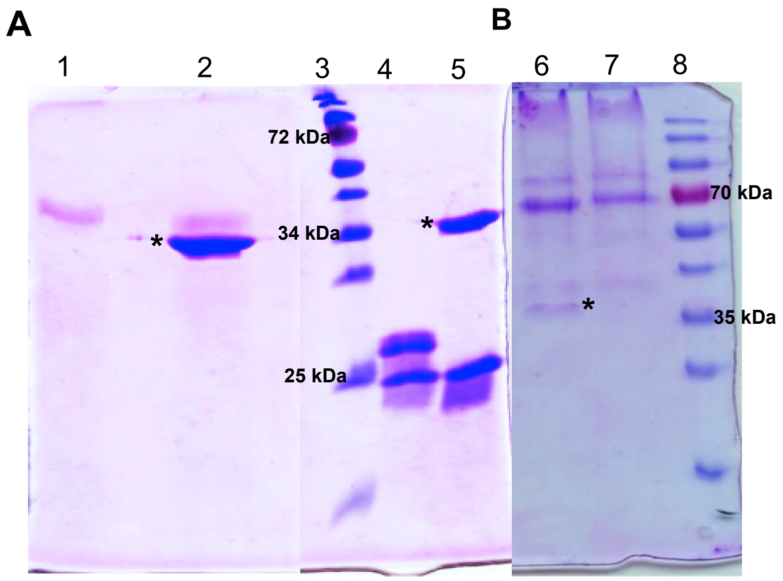

Figure S5. SDS-PAGE after PNGase F treatment of the recombinant Md1RIP (**A**) or Md2RIP (**B**). Lanes 1, 4 and 7: untreated Md1RIP, RNAse B and Md2RIP; lanes 2, 5 and 6: Md1RIP, RNAse B and Md2RIP treated with PNGase F. Lanes 3 and 8: protein ladder (Fermentas). In each well, 2 μg protein was loaded. The position of the polypeptide corresponding to PNGase F is indicated with an asterisk.

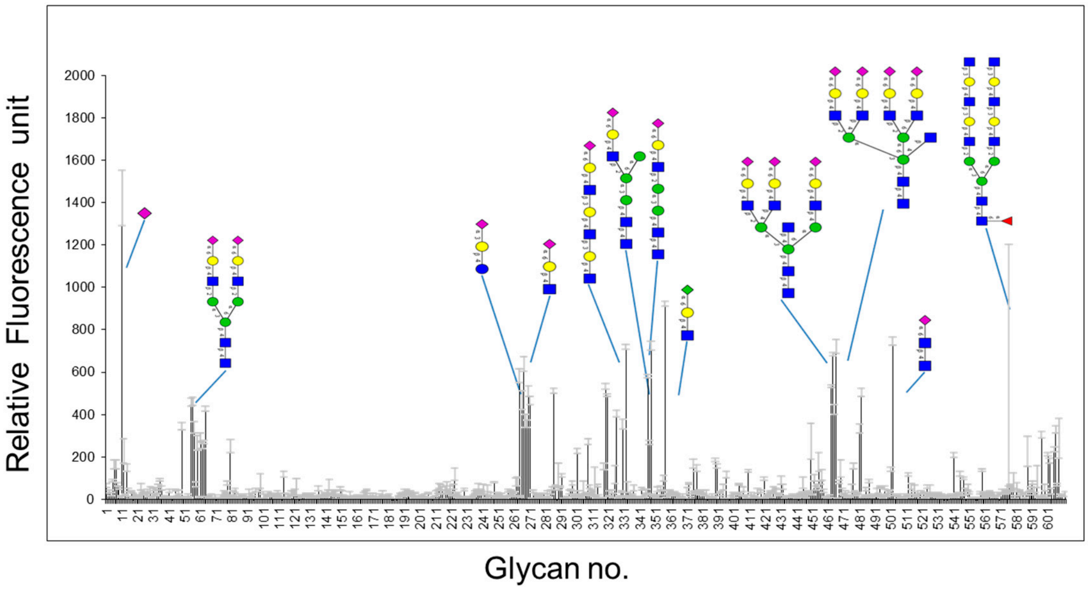

Figure S6. Glycan array analysis of recombinant Md2RIP at 300 μg/ml. The Consortium for Functional Glycomics website (http://www.functionalglycomic.org) supports the complete raw data for all the proteins. Sugar code used: green circles represent mannose residues, yellow circles indicate Gal, blue squares indicate GlcNAc residues, red triangles show fucose, purple diamonds indicate NeuAc and green diamonds indicate KDN.

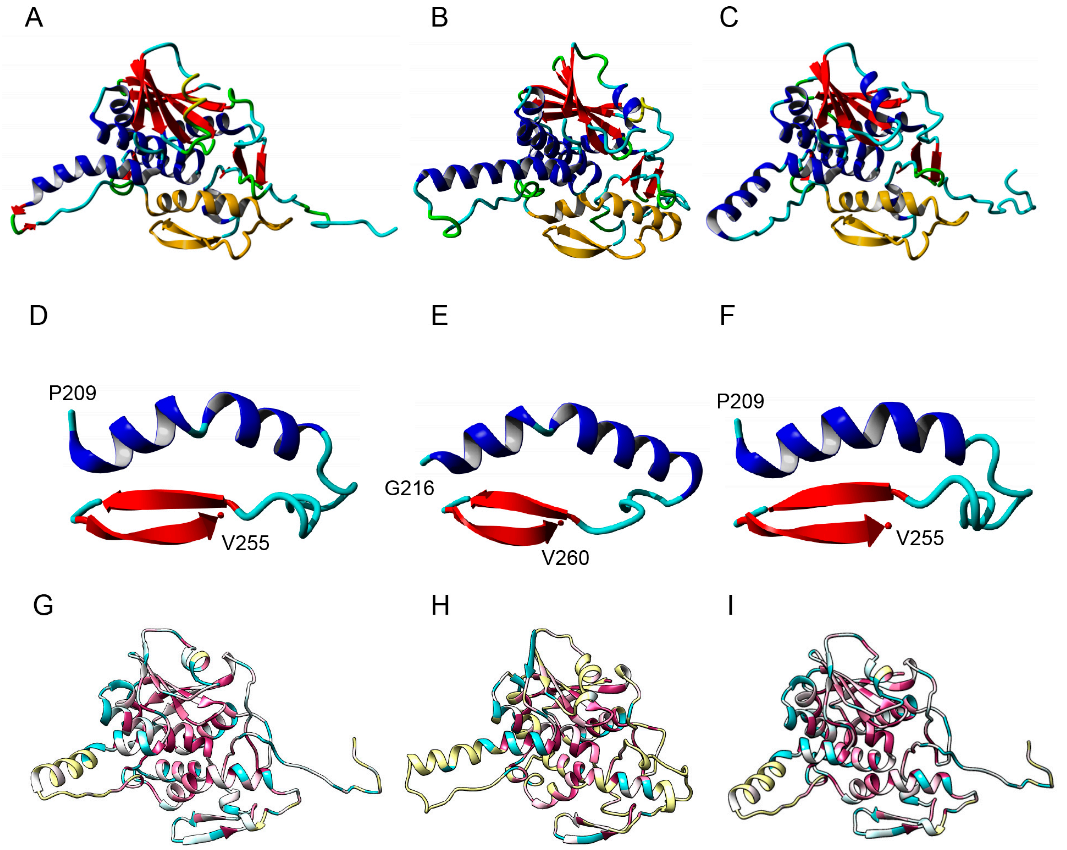

Figure S7. Ribbon diagrams of type 1 RIP from apple (**A**), peach (**B**) and pear (**C**), showing the α-helix-β-hairpin structure (colored orange) located in the C-terminal region of the RIPs. Structural similarity of the α-helix-β-hairpin structure of type 1 RIPs of apple (**D**), peach (**E**) and pear (**F**). Conservation of the secondary structural features of type 1 RIP of apple (**G**), peach (**H**) and pear (**I**). The code-colored conservation scale used by ConSurf is as follows:

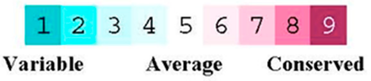

A

```
BE27  201 WSKISEGIRKA---VKKVIS-PPIELVNASNGKWTVNQV 235
Apple 220 WSNLSEQIQMVPANQLLFINNGSVQIRKADNSIVLVKSV 258
Peach 226 WSDLSEQIQRAQQRNETGFD-RTIVLHNVGNERREVNSV 263
Pear  220 WSNLSEQIQMVPANQLLFINNGSVQIRKADNSIVLVKSV 258
```

B

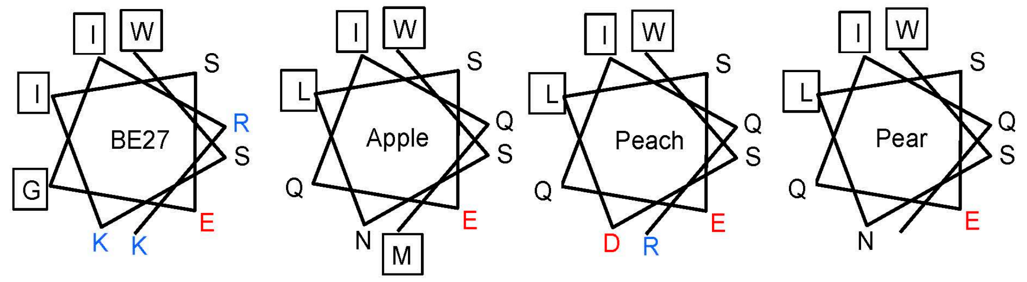

Figure S8. (**A**) Alignment of the α-helix-β-hairpin motif sequences from BE27 and type 1 RIPs from apple, peach and pear; (**B**) Helical wheel drawing of the helices of BE27, apple, peach and pear. The amino acid charge is indicated in red (negative), black (neutral) and blue (positive). Hydrophobic amino acids are boxed [3,4].

Table S1. List of genes encoding RIPs from Rosaceae.

Species	Accession Number	Size	Location in the Genome	Signal Sequence	Sequence Database Source
**Type 1 RIP**					
Malus domestica	MDP0000918923	301 aa	unanchored	No	Genome database for Rosaceae
	MDP0000223290	314 aa	unanchored	No	
	MDP0000134012	306 aa	Chr3	No	
Pyrus communis	PCP001408.1	301 aa	unanchored	No	
	PCP026877.1	276 aa	unanchored	No	
	PCP011148.1	283 aa	unanchored	No	
Pyrus bretschneideri	XP_009346751.1	278 aa	unanchored	No	NCBI database
	XP_009346753.1	246 aa	unanchored	No	
	XP_009375039.1	278 aa	unanchored	No	
	XP_009374990.1	280 aa	unanchored	No	
Prunus mume	XP_008243880.1	201 aa	unanchored	No	
	XP_008243881	304 aa	unanchored	No	
	XP_016652174.1	304 aa	unanchored	No	
	XP_016652175.1	294 aa	unanchored	No	
Prunus persica	ppa009409mg	294 aa	unanchored	No	Phytozome database
	ppa009637mg	283 aa	unanchored	No	
**Type 2 RIP**					
Malus domestica	MDP0000711911	549 aa	Chr8	Yes	Genome database for Rosaceae
Pyrus communis	PCP031611	547 aa	unanchored	Yes	

Table S2. Overview of the top 30 glycans interacting with the Md2RIP and comparative analysis with SNA-I. The glycan with the highest relative fluorescence unit (RFU) is assigned a value of 100. The rank is the percentile ranking. NA: not available in this glycan array version.

Glycan No.	Structure	Md2RIP 300 μg/mL		SNA-I 0.1 μg/mL	
		RFU	Rank	RFU	Rank
11	Neu5Acβ-Sp8	1421	100	25	0.09
357	KDNα2-6Galβ1-4GlcNAc-Sp0	922	64.9	30	0.11
502	Neu5Acα2-6GalNAcβ1-4(6S)GlcNAcβ-Sp8	746	52.5	NA	NA
348	Neu5Acα2-6Galβ1-4GlcNAcβ1-2Manα1-3Manβ1-4GlcNAcβ1-4GlcNAc-Sp12	724	51.0	17952	65.01
466	Neu5Acα2-6Galβ1-4GlcNAcβ1-6(Neu5Acα2-6Galβ1-4GlcNAcβ1-2)Manα1-6(GlcNAcβ1-4)(Neu5Acα2-6Galβ1-4GlcNAcβ1-4(Neu5Acα2-6Galβ1-4GlcNAcβ1-2)Manα1-3)Manβ1-4GlcNAcβ1-4GlcNAcβ-Sp21	721	50.8	NA	NA
332	Neu5Acα2-6Galβ1-4GlcNAcβ1-3Galβ1-4GlcNAcβ1-3Galβ1-4GlcNAcβ-Sp0	719	50.6	24563	88.95
464	Neu5Acα2-6Galβ1-4GlcNAcβ1-4Manα1-6(GlcNAcβ1-4)(Neu5Acα2-6Galβ1-4GlcNAcβ1-4 (Neu5Acα2-6Galβ1-4GlcNAcβ1-2)Manα1-3)Manβ1-4GlcNAcβ1-4GlcNAcβ-Sp21	683	48.1	NA	NA
267	Neu5Acα2-6Galβ1-4(6S)GlcNAcβ-Sp8	638	44.9	14288	51.74
576	GlcNAcβ1-3Galβ1-4GlcNAcβ1-3Galβ1-4GlcNAcβ1-2Manα1-6(GlcNAcβ1-3Galβ1-4GlcNAcβ1-3Galβ1-4GlcNAcβ1-2Manα1-3)Manβ1-4GlcNAcβ1-4(Fucα1-6)GlcNAcβ-Sp24	633	44.6	NA	NA
264	Neu5Acα2-3Galβ1-4Glcβ-Sp8	582	40.9	18033	26.30
346	Manα1-6(Neu5Acα2-6Galβ1-4GlcNAcβ1-2Manα1-3)Manβ1-4GlcNAcβ1-4GlcNAc-Sp12	581	40.9	NA	NA
319	Galβ1-4GlcNAcβ1-2Manα1-6(Neu5Acα2-6Galβ1-4GlcNAcβ1-2Mαnα1-3)Manβ1-4GlcNAcβ1-4GlcNAcβ-Sp12	533	37.5	22956	83.13
463	Neu5Acα2-6Galβ1-4GlcNAcβ1-2Manα1-6(GlcNAcβ1-4)(Neu5Acα2-6Galβ1-4GlcNAcβ1-2Manα1-3)Manβ1-4GlcNAcβ1-4GlcNAcβ-Sp21	533	37.5	NA	NA
286	Neu5Gcα2-6Galβ1-4GlcNAcβ-Sp0	510	35.9	13790	49.94
270	Neu5Acα2-6Galβ1-4GlcNAcβ1-3Galβ1-4(Fucα1-3)GlcNAcβ1-3Galβ1-4(Fucα1-3)GlcNAcβ-Sp0	510	35.9	20964	75.91
482	Neu5Acα2-6Galβ1-4GlcNAcβ1-2Manα1-6(Neu5Acα2-6Galβ1-4GlcNAcβ1-2Manα1-3)Manβ1-4GlcNAcβ1-4(Fucα1-6)GlcNAcβ-Sp24	504	35.5	NA	NA
320	GlcNAcβ1-2Manα1-6(Neu5Acα2-6Galβ1-4GlcNAcβ1-2Manα1-3)Manβ1-4GlcNAcβ1-4GlcNAcβ-Sp12	493	34.7	14464	52.38
271	Neu5Acα2-6Galβ1-4GlcNAcβ1-3Galβ1-4GlcNAcβ-Sp0	467	32.9	25668	92.95
266	Neu5Acα2-6GalNAcβ1-4GlcNAcβ-Sp0	466	32.8	29	0.11
56	Neu5Acα2-6Galβ1-4GlcNAcβ1-2Manα1-6(Neu5Acα2-6Galβ1-4GlcNAcβ1-2Man-α1-3)Manβ1-4GlcNAcβ1-4GlcNAcβ-Sp21	462	32.5	NA	NA
55	Neu5Acα2-6Galβ1-4GlcNAcβ1-2Manα1-6(Neu5Acα2-6Galβ1-4GlcNAcβ1-2Manα1-3)Manβ1-4GlcNAcβ1-4GlcNAcβ-Sp12	458	32.3	18898	66.44
64	Fucα1-2Galβ1-3GalNAcβ1-4(Neu5Acα2-3)Galβ1-4Glcβ-Sp9	429	30.2	19	0.07
465	Neu5Acα2-6Galβ1-4GlcNAcβ1-6(Neu5Acα2-6Galβ1-4GlcNAcβ1-2)Manα1-6(GlcNAcβ1-4) (Neu5Acα2-6Galβ1-4GlcNAcβ1-2Manα1-3)Manβ1-4GlcNAcβ1-4GlcNAcβ-Sp21	424	29.8	NA	NA
326	Neu5Acα2-3Galβ1-4GlcNAcβ1-2Manα1-6(Neu5Acα2-6Galβ1-4GlcNAcβ1-2Manα1-3)Manβ1-4GlcNAcβ1-4GlcNAcβ-Sp12	406	28.5	10	0.04
268	Neu5Acα2-6Galβ1-4GlcNAcβ-Sp0	370	28.6	27613	100
269	Neu5Acα2-6Galβ1-4GlcNAcβ-Sp8	355	26.0	14288	51.74
330	Neu5Acα2-6Galβ1-4GlcNAcβ1-3Galβ1-3GlcNAcβ-Sp0	353	25	21014	76.10
49	Neu5,9Ac2α2-6Galβ1-4GlcNAcβ-Sp8	344	24.9	21953	79.50
57	Neu5Acα2-6Galβ1-4GlcNAcβ1-2Manα1-6(Neu5Acα2-6Galβ1-4GlcNAcβ1-2Manα1-3)Manβ1-4GlcNAcβ1-4GlcNAcβ-Sp24	339	24.2	NA	NA
481	Neu5Acα2-6Galβ1-4 GlcNAcβ1-6(Neu5Acα2-6Galβ1-4GlcNAcβ1-3)GalNAcα-Sp14	334	23.9	NA	NA

Table S3. Geometric and thermodynamic qualities of the RIP models built by homology modeling (* since ANOLEA works with 3D structures, both the numbering of residues (*) and the number of residues (**), refer to the models built for type 1 and type 2 RIPs from Rosaceae).

Models	Residues * out of the Allowed Areas in the Ramachandran Plot	Residues with Values over the Threshold in the ANOLEA Plot	QMEAN Value
Type 1 RIP apple	D44, A91, F101, N233 (4)	15 out of 292 residues **	0.60
Type 1 RIP peach	N9, D44, D81, V151, T342 (5)	11 out of 294 residues **	0.57
Type 1 RIP pear	A91, F101 (2)	5 out of 292 residues **	0.58
Type 2 RIP apple	K273, E344, S353 (3)	15 out of 538 residues **	0.56
Type 2 RIP pear	D99, H142, A245, N250, I252, E324, Y411, D420 (8)	11 out of 528 residues **	0.57

References

1. Kaku, H.; Kaneko, H.; Minamihara, N.; Iwata, K.; Jordan, E.T.; Rojo, MA.; Minami-Ishii, N.; Minami, E.; Hisajima, S.; Shibuya, N. Elderberry bark lectins evolved to recognize Neu5Acα2,6Gal/GalNAc sequence from a Gal/GalNAc binding lectin through the substitution of amino-acid residues critical for the binding to sialic acid. *Biochem. J.* **2007**, *142*, 393–401.
2. Hu, D.; Tateno, H.; Kuno, A.; Yabe, R.; Hirabayashi, J. Directed evolution of lectins with sugar-binding specificity for 6-sulfogalactose. *J. Biol. Chem.* **2012**, *287*, 20313–20320.
3. Citores, L.; Iglesias, R.; Gay, C.; Ferreras, J.M. Antifungal activity of the ribosome-inactivating protein BE27 from sugar beet (*Beta vulgaris* L.) against the green mould *Penicillium digitatum*. *Mol. Plant Pathol.* **2016**, *17*, 261–271.
4. Iglesias, R.; Citores, L.; Ragucci, S.; Russo, R.; di Maro, A.; Ferreras, J.M. Biological and antipathogenic activities of ribosome-inactivating proteins from *Phytolacca dioica* L. *BBA-Gen. Subjects* **2016**, *1860*, 1256–1264.
